# Down, then up: non-parallel genome size changes and a descending chromosome series in a recent radiation of the Australian allotetraploid plant species, *Nicotiana* section *Suaveolentes* (Solanaceae)

**DOI:** 10.1093/aob/mcac006

**Published:** 2022-01-13

**Authors:** Mark W Chase, Rosabelle Samuel, Andrew R Leitch, Maïté S Guignard, John G Conran, Felipe Nollet, Paul Fletcher, Aljaž Jakob, Luiz A Cauz-Santos, Gabriel Vignolle, Steven Dodsworth, Maarten J M Christenhusz, Maria Teresa Buril, Ovidiu Paun

**Affiliations:** Royal Botanic Gardens, Kew, Richmond TW9 3DS, UK; Department of Environment and Agriculture, Curtin University, Perth, Western Australia, Australia; Department of Botany and Biodiversity Research, University of Vienna, Rennweg 14, A-1030 Vienna, Austria; Department of Botany and Biodiversity Research, University of Vienna, Rennweg 14, A-1030 Vienna, Austria; School of Biological and Chemical Sciences, Queen Mary University of London, Mile End Road, London E1 4NS, UK; Royal Botanic Gardens, Kew, Richmond TW9 3DS, UK; ACEBB & SGC, School of Biological Sciences, The University of Adelaide, SA 5005Australia; Universidade Federal Rural de Pernambuco, Centro de Ciências Biológicas, Departamento de Botânica, Rua Manuel de Medeiros, S/N, Dois Irmãos, 52171-900 Recife, Pernambuco, Brazil; School of Biological and Chemical Sciences, Queen Mary University of London, Mile End Road, London E1 4NS, UK; Department of Botany and Biodiversity Research, University of Vienna, Rennweg 14, A-1030 Vienna, Austria; Department of Botany and Biodiversity Research, University of Vienna, Rennweg 14, A-1030 Vienna, Austria; Department of Botany and Biodiversity Research, University of Vienna, Rennweg 14, A-1030 Vienna, Austria; School of Biological Sciences, University of Portsmouth, Portsmouth PO1 2DY, UK; Department of Environment and Agriculture, Curtin University, Perth, Western Australia, Australia; ACEBB & SGC, School of Biological Sciences, The University of Adelaide, SA 5005Australia; Department of Botany and Biodiversity Research, University of Vienna, Rennweg 14, A-1030 Vienna, Austria

**Keywords:** Allotetraploid evolution, Australian endemics, C-value, diploidization, dysploidy, epigenetics, model organism, *Nicotiana benthamiana*, *Nicotiana* sect. *Suaveolentes*, polyploidy, Solanaceae, WGD

## Abstract

**Background and Aims:**

The extent to which genome size and chromosome numbers evolve in concert is little understood, particularly after polyploidy (whole-genome duplication), when a genome returns to a diploid-like condition (diploidization). We study this phenomenon in 46 species of allotetraploid *Nicotiana* section *Suaveolentes* (Solanaceae), which formed <6 million years ago and radiated in the arid centre of Australia.

**Methods:**

We analysed newly assessed genome sizes and chromosome numbers within the context of a restriction site-associated nuclear DNA (RADseq) phylogenetic framework.

**Key Results:**

RADseq generated a well-supported phylogenetic tree, in which multiple accessions from each species formed unique genetic clusters. Chromosome numbers and genome sizes vary from *n* = *2x* = 15 to 24 and 2.7 to 5.8 pg/1C nucleus, respectively. Decreases in both genome size and chromosome number occur, although neither consistently nor in parallel. Species with the lowest chromosome numbers (*n* = 15–18) do not possess the smallest genome sizes and, although *N. heterantha* has retained the ancestral chromosome complement, *n* = *2x* = 24, it nonetheless has the smallest genome size, even smaller than that of the modern representatives of ancestral diploids.

**Conclusions:**

The results indicate that decreases in genome size and chromosome number occur in parallel down to a chromosome number threshold, *n* = 20, below which genome size increases, a phenomenon potentially explained by decreasing rates of recombination over fewer chromosomes. We hypothesize that, more generally in plants, major decreases in genome size post-polyploidization take place while chromosome numbers are still high because in these stages elimination of retrotransposons and other repetitive elements is more efficient. Once such major genome size change has been accomplished, then dysploid chromosome reductions take place to reorganize these smaller genomes, producing species with small genomes and low chromosome numbers such as those observed in many annual angiosperms, including *Arabidopsis*.

## INTRODUCTION

Chromosome number and genome size changes in angiosperms have been poorly explored in a phylogenetic context at the species level, especially in a post-polyploid (whole-genome duplication, WGD) context. Aside from WGD, it has long been known that amplification and deletion of highly repetitive DNA, especially retroelements, are mechanisms by which genome size (C-value) change occurs (Bennetzen and Kellogg, 1997; Wang *et al.*, 2021). Genome size is thus a compromise between the activities of various mechanisms in the ancestry of a clade, including intensity of (retro)transposition and frequency of WGDs, that increase genome size and recombination-related processes that purge portions of the genome (Hawkins *et al.*, 2009; Grover and Wendel, 2010; Michael, 2014).

In general, the packaging of chromatin, DNA break repair and activity of (retro)transposons and other repetitive elements is under epigenetic control (Fedoroff, 2012), probably influencing changes in genome size (through increases/decreases in repeat numbers and structure), frequency and occurrence of chromosome rearrangements and genome stability (Van der Knaap *et al.*, 2004; Schubert and Vu, 2016). In previous studies, a positive correlation between rates of genome size evolution and speciation across the angiosperm phylogenetic tree has been shown (Leitch and Leitch, 2008; Puttick *et al.*, 2015), but there has been no general relationship demonstrated between genome size, direction of chromosome number change and speciation. Angiosperm genome sizes have also been shown not to be directly proportional to ploidy (Leitch and Bennett, 2004; Hufton and Panopoulou, 2009; Rupp *et al.*, 2010; Carta *et al.*, 2020).

In some groups, e.g. *Nicotiana* section *Suaveolentes* (approx. 49 species, the subject of this study), high rates of genome size change and chromosome structural changes are correlated with a range of phenomena (Oliver and Greene, 2009; Oliver *et al.*, 2009) that are putatively promoting the high rates of speciation detected (Clarkson *et al.*, 2017). A change in chromosome number is often more complex than simply fusing two into one (or vice versa) and in most groups involves multiple chromosome segment exchanges (Mandáková and Lysak, 2008); presumably these rearrangements directly alter linkage among genes in the segments that have been reorganized (Morjan and Rieseberg, 2004; Ortiz-Barrientos *et al.*, 2016; Merot *et al.*, 2020). Reduced recombination through the formation of new, often larger, linkage groups (i.e. fewer chromosomes) is hypothesized to protect highly advantageous allele combinations in incipient species (Stebbins, 1950), promoting local adaptation and increasing net diversification (Potter *et al.*, 2017). Although putatively advantageous in this context, fewer chromosomes and the resulting lower rates of recombination could lead to increasing levels of retrotransposon activity because their control is due to recombination-related processes, as noted above, which could result in larger genomes. It is in this context that we have focused this study, to study chromosome and genome size divergence over a large range of chromosome numbers (*n* = 15–24) post-WGD in the framework of a nearly complete species-level phylogenetic analysis of *Nicotiana* sect. *Suaveolentes* (Goodspeed, 1954; Purdie *et al*., 1982; Chase *et al.*, 2018*a*).

*Nicotiana* sect. *Suaveolentes* has been studied for a long time, starting with the chromosome studies and monograph of Goodspeed (1954), who concluded correctly that the section is ancestrally allotetraploid. Many molecular studies have now demonstrated that they have a single origin via hybridization between two South American diploid species, both with *n* = 12, one likely to have been itself a diploid hybrid (Chase *et al.*, 2003; Clarkson *et al*., 2004, 2010; Kelly *et al.*, 2013; Schiavinato *et al.*, 2019; Dodsworth *et al.*, 2020*a*), leading to an ancestral *Nicotiana* sect. *Suaveolentes* species with *n* = 24. Divergence of the section has probably involved multiple dysploid reductions to give the current range *n* = 15–24.

**Fig. 1.**
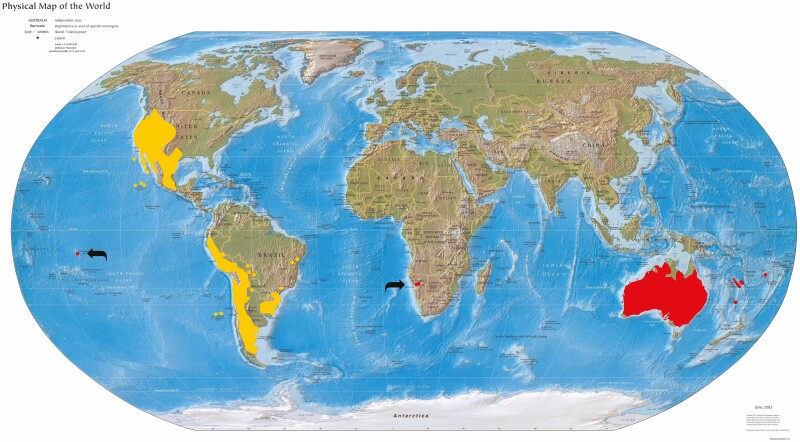
Global distribution of *Nicotiana* with distribution of *N.* sect. *Suaveolentes* in red and that of the New World species in yellow. Map base from Wikimedia Commons.

The common ancestor of *Nicotiana* sect. *Suaveolentes* arose 5–6 Mya (million years ago; Clarkson *et al.*, 2017; Schiavinato *et al.*, 2019) in central western South America, and then its descendants dispersed widely (Fig. 1), resulting today in species in Africa (one, in Namibia, *N. africana*), Australia (approx. 46 species, especially numerous in the most arid central regions), New Caledonia (one species, *N. fragrans* shared with other Pacific islands, plus another, *N. forsteri*, also in eastern Australia) and several islands in French Polynesia (one species, *N. fatuhivensis*). None of the species of *Nicotiana* sect. *Suaveolentes* is known from the Americas. Extant species with the ancestral (or nearly so) chromosome number, *n* = 23 or 24, are found in Africa, wetter northern and eastern Australia and the Pacific islands (the chromosome number of *N. fatuhivensis* is unknown because living material has not been available for cytological study).

About 2 Mya, the common ancestor of the species-rich ‘core group’ of *Nicotiana* sect. *Suaveolentes* invaded the already well-established arid centre of Australia (which became as dry as today about 7 Mya; Byrne *et al.*, 2008) and diversified to produce the currently recognized plethora of Australian species (approx. 49; Chase *et al*., 2018*a*, 2021*a*). Their putative recent origin has resulted in standard molecular markers (e.g. Chase *et al.*, 2003; Clarkson *et al.*, 2010; Marks *et al.*, 2011; Bally *et al.*, 2021) exhibiting low levels of variability, making previous phylogenetic inferences both tentative and weakly supported. In addition, Dodsworth *et al.* (2020*b*) found high levels of ancestral polymorphisms that made whole plastome DNA sequences unreliable for estimating species relationships in this section. Accordingly, this study examines the phylogenetics of 46 (out of 49) species of *Nicotiana* sect. *Suaveolentes* using restriction site-associated DNA sequencing data (RADseq; Baird *et al.*, 2008), which has worked well to resolve other recently diverged groups (Cruaud *et al.*, 2014; Paun *et al.*, 2016), including some older than these species (e.g. Heckenhauer *et al.*, 2018; Brandrud *et al*., 2019, 2020; Wagner *et al.*, 2020).

Using this robust nuclear phylogenetic tree as framework, we examine the relationship between chromosome number and genome size change, and hypothesize that because both genome size and chromosome number changes are impacted by epigenetic phenomena [e.g. the activity of (retro)transposable elements, mechanisms of DNA repair and condensation of chromatin], it is possible that they will exhibit similar levels/directions of change across the phylogenetic tree. Chromosome numbers typically descend post-WGD in herbaceous species, and genome size shrinkage has also been recorded, and thus we expect to see both occurring among the species of *Nicotiana* sect. *Suaveolentes.* We further hypothesize that if chromosome number falls far enough, genome size might start to increase due to the lower number of chiasmata formed during meiosis and, hence, lower levels of recombination. This pattern has been observed previously (Chase *et al.*, 2005; Lysak *et al.*, 2009), but not demonstrated with complete species-level sampling in a group exhibiting simultaneous post-polyploid genome size and extensive chromosome number change.

## MATERIALS AND METHODS

### Taxon sampling, plant material and deposition of vouchers

**Fig. 2.**
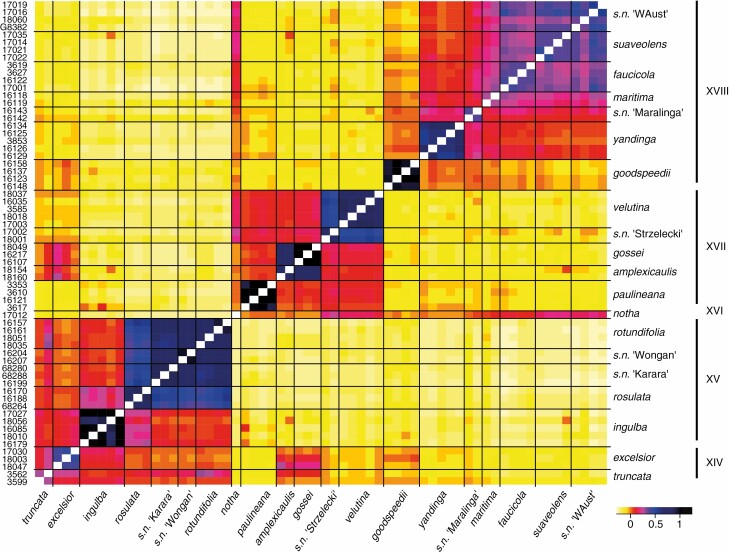
Co-ancestry heatmap3, constructed based on genotype likelihoods. Darker tones represent higher pairwise relatedness; estimates on the diagonal have been excluded.

**Fig. 3.**
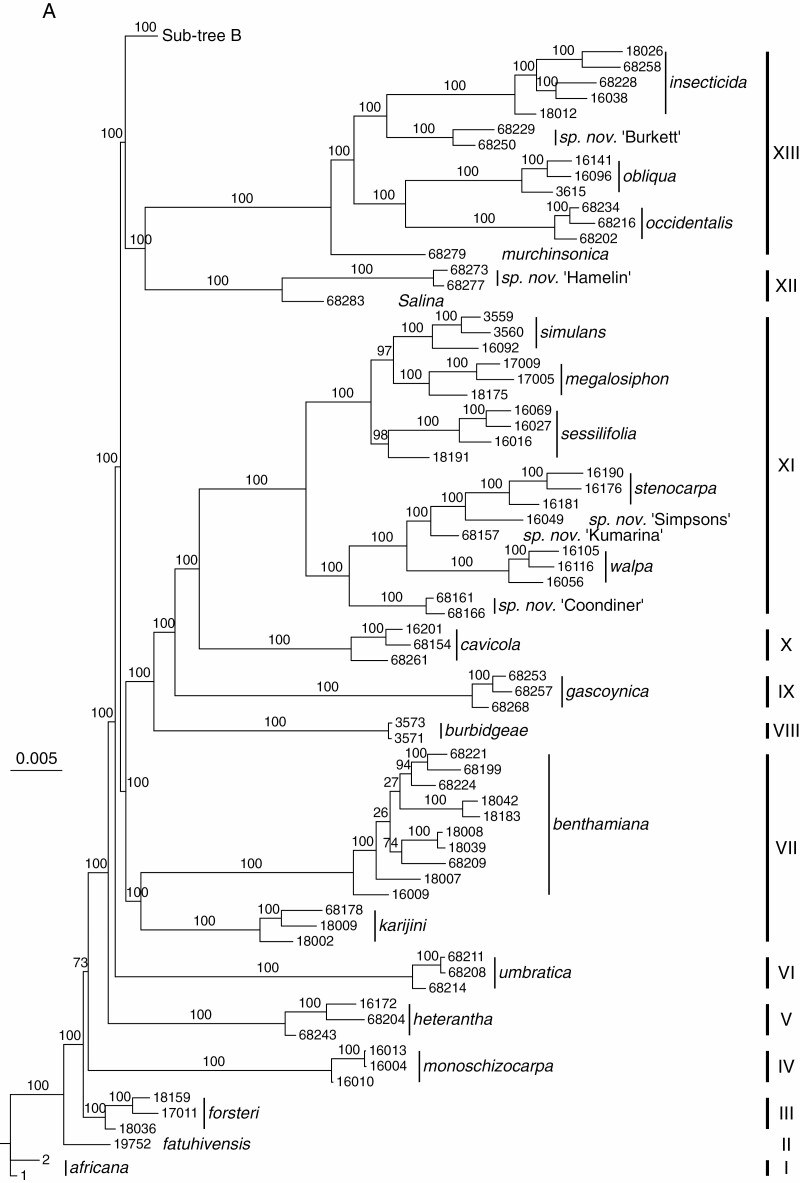

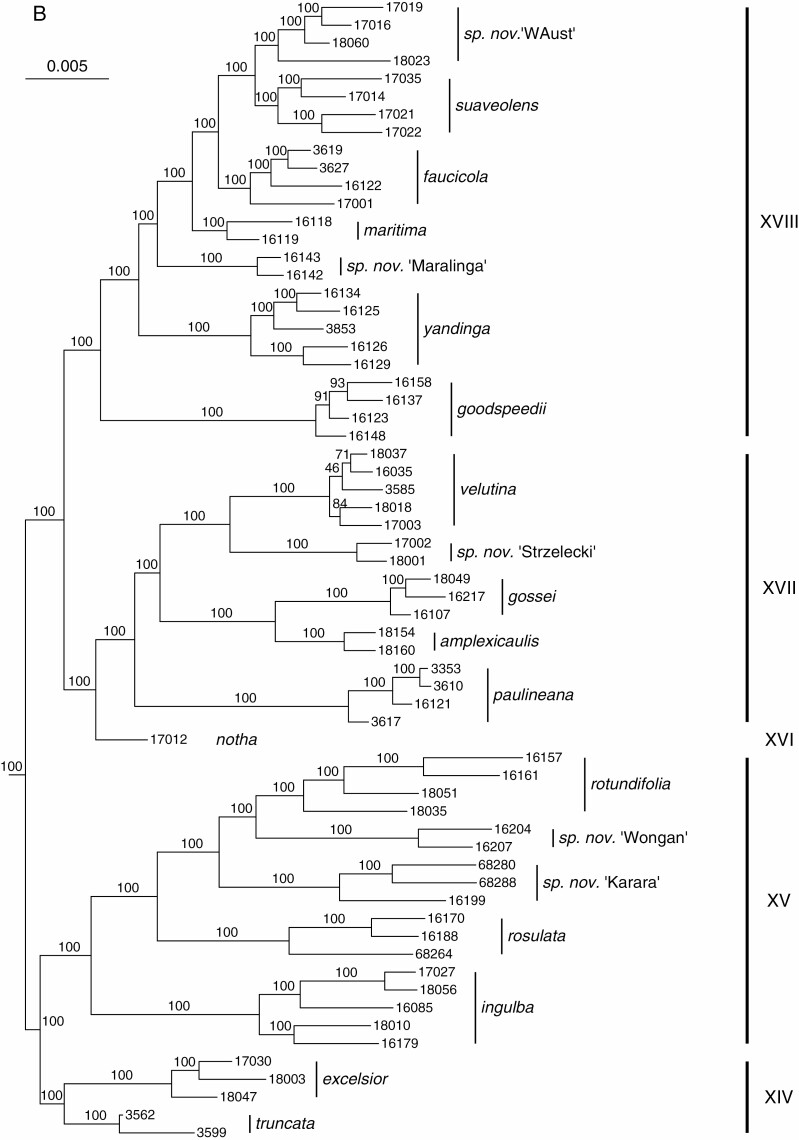
RADseq tree, sub-tree A (top) and sub-tree B (bottom). RAxML-derived phylogenetic tree based on 457 382 single nucleotide polymorphisms (SNPs).

**Fig. 4.**
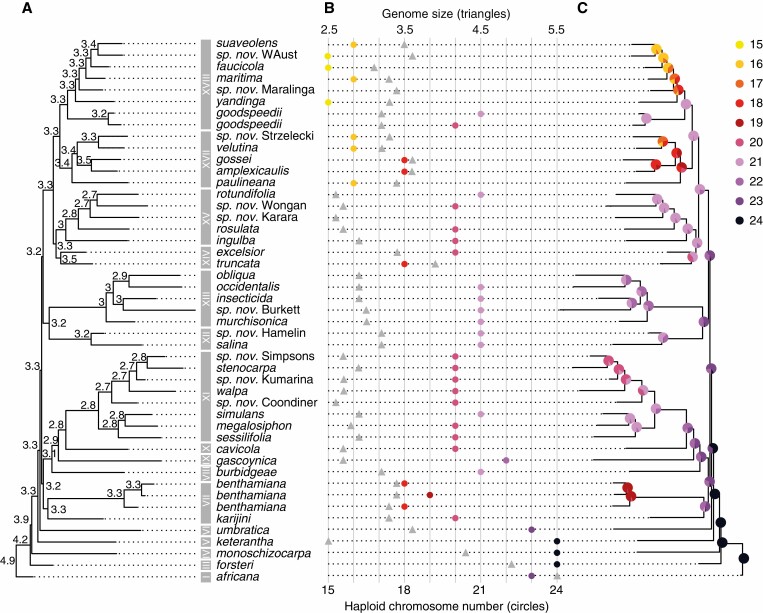
Present state and ancestral reconstruction of chromosome number and genome size. (A) The summary tree of species relationships (with locality names for undescribed new species, as in Fig. 3), as estimated with ChromEvol with chromosome number increases prohibited. (B) Genome sizes (triangles, upper *x*-scale) and haploid chromosome number (circles, lower X-scale). (C) Genome size evolution as estimated using BayesTree using the summary tree of species relationships. Chromosome number according to the embedded colour legend.

Our sampling of phylogenetic data included 137 individuals, representing 46 putative species, with the aim to analyse multiple accessions per species, including samples from across the geographic and morphological ranges of each species; however, for some taxa, this was not possible (e.g. *N. fatuhivensis* and *N. murchisonica*; Table 1). As we conducted the RADseq sampling, several accessions unexpectedly did not fall with others of the species to which we had assigned them initially, based on their morphological features as assessed in the field (e.g. *N. sp. nov*. Karara). Species in the group appear to have a relatively high degree of phenotypic plasticity depending on patterns of rainfall and, in some cases, we initially assigned samples mistakenly to the wrong species. We have already described some of the most obvious of these misplaced accessions as new (Chase *et al*., 2018*a*, 2021*a*), and other such treatments are in progress. The new species that require more research before they can be described are labelled here as ‘*sp. nov*.’ with a locality name (e.g. *sp. nov*. Karara; Figs 2–4).

**Table 1.** Accession voucher numbers, provenance data, genome size estimates and chromosome numbers

Species name in *Nicotiana*	Voucher number*	Latitude/longitude (S and E; degrees, minutes, seconds)	Provenance (brief locality name, all Australia, except where noted otherwise)	Genome size estimate (pg/1C)	Chromosome number (2*n*; count here unless literature reference provided)
*africana*	TW6		Namibia	5.5	46, Tatemichi (1990); Kitamura *et al.* (2005)
*africana*	TW6		Namibia	5.4	
*amplexicaulis*	18160	24, 19, 49; 147, 26, 15	Nogoa River, Queensland	3.6	36 here; 36, Tatemichi (1990)
*amplexicaulis*	18154	25, 10, 37; 148, 33, 56	Moolayember Gap, Queensland		
*benthamiana*	68199	20, 50, 22; 117, 8, 13	Roebourne, Western Australia	3.1	
*benthamiana*	68221	21, 3, 12; 116, 15, 12	Mardie, Western Australia	3.4	36
*benthamiana*	68224	21, 39, 39; 116, 16, 30	Pannawonica, Western Australia	3.3	
*benthamiana*	18183	21, 6, 47; 139, 48, 54	Duchess/Mount Isa Road, Queensland	3.4	
*benthamiana*	18042 (Wannan 5860; BRI AQ0855406)	16, 40, 43; 144, 12, 29	Bellevue, Queensland		
*benthamiana*	18039 (Bean 25412; BRI AQ735848)	22, 52, 55; 119, 14, 9	Weeli Wolli Creek, Western Australia	3.4	
*benthamiana*	18008 (Latz 18092; PERTH 8305579)	22, 2, 25.08; 129, 20, 14.1	Lake Mackay, Northern Territory	3.4	38 here; 38, Tatemichi (1990)
*benthamiana*	68209	21, 33, 44; 119, 19, 14	Woodstock–Marble Bar Road, Western Australia	3.3	36
*benthamiana*	18007 (Muir 1023; PERTH 8610134),	24, 4, 9.8; 123, 9, 58	Little Sandy Desert, Western Australia	3.3	
*benthamiana*	16009	15, 36, 45; 131, 8, 57	Victoria River Crossing, Northern Territory	3.4	
*benthamiana* †	68200	20, 53, 19; 117, 20, 26	Wittenoom, North West Coastal Highway, Western Australia	3.4	
*benthamiana* †	68171	23, 17, 3; 119, 39, 30	Silent Gorge, approx. 10 km west of Newman, Western Australia		38
*benthamiana* †	68174	23, 2, 27; 118, 51, 4	Mt Robinson, along trail into gorge, Western Australia	3.4	
*benthamiana* †	18181	21, 23, 8; 139, 49, 53	Duchess/Dajarra Road, 5 km south-west of Duchess, Queensland	3.4	
*burbidgeae*	Conran 3571 (AD)	26, 40, 21; 135, 37, 16	Mount Sarah, South Australia	3.2	42, Symon (1984)
*burbidgeae*	Conran 3573 (AD)	26, 33, 35; 135, 31, 11	South of Dalhousie Springs, South Australia	3.2	
*cavicola*	68154	26, 34, 56; 118, 39, 7	Meekatharra, Western Australia	2.7	40 here; 40, Williams (1975); 46; Burbidge (1960); 46, Tatemichi (1990)
*cavicola*	16201	29, 2, 54; 117, 19, 17	Thundelarra Station, Western Australia	2.7	
*cavicola*	68261	25, 17, 35; 115, 42, 27	Congo Creek, Western Australia		
*excelsior*	18003 (Vonow 3206; AD 201220)	24, 53, 17; 128, 46, 2	Kutjuntari Rockhole, Western Australia	3.4	
*excelsior*	17030	25, 17, 57; 130, 44, 2	Kata-Tjuta, Northern Territory	3.4	40 here; 38, Tatemichi (1990)
*excelsior*	18047 (Latz 25867; NT D0198637)	26, 5, 9; 132, 12, 35	Ernabella, South Australia		
*fatuhivensis*	Wood 10529 (NTBG)	8, 55, 14; 139 32 49	Ua Huka, French Polynesia		
*faucicola*	Conran 3627 (AD)	31, 24, 14; 138, 42, 31	Flinders Ranges Way, South Australia		
*faucicola*	Conran 3619 (AD)	31, 25, 5; 138, 33, 40	Bunyeroo Gorge, South Australia		
*faucicola*	16122	33, 1, 28; 138, 6, 10	Telowie Gorge, South Australia		30
*faucicola*	17001	33, 50, 1.84; 139, 1, 53.57	Burra Gorge, South Australia	3.1	
*forsteri*	17011	31, 2, 54.6; 153, 3, 53.3	Hat Head, New South Wales	4.9	
*forsteri*	18159	23, 9, 22; 150, 27, 10	Rockhampton, New South Wales	4.9	
*forsteri*	18036 (Bean 28592; BRI AQ0820646)	26, 28, 11; 152, 20, 23	Gallangowan State Forest, Queensland		
*forsteri* †	18030 (Forster 41986; BRI AQ0837934)	26, 3, 59; 151, 36, 46	Woroon National Park, Queensland	4.9	
*forsteri* †	18063 (Bean 30653; BRI AQ0822714)	21, 25, 36; 148, 33, 56	Marling Spike, Homevale National Park, Queensland		48 here; 48, Tatemichi (1990); Kitamura *et al.* (2005)
*gascoynica*	68257	24, 45, 21; 114, 8, 10	Rocky Pool, Western Australia	2.7	
*gascoynica*	68253	24, 49, 41; 113, 46, 12	Gascoyne River Bridge, Western Australia		44
*gascoynica*	68268	25, 45, 29; 114, 16, 41	Wooramel River Bridge, Western Australia	2.7	
*goodspeedii*	16137	31, 54, 48; 132, 29, 18	Fowlers Bay, South Australia	3.2	40 here; 40, Tatemichi (1990)
*goodspeedii*	16158	31, 42, 28; 121, 41, 12	Kalgoorlie–Norseman Road, Western Australia		
*goodspeedii*	16123	33, 17, 25; 137, 17, 50	Moonabie Beach, South Australia		
*goodspeedii*	16148	31, 36, 19; 130, 45, 18	Nullarbor Roadhouse, South Australia		42 here
*gossei*	16217	23, 40, 37; 133, 43, 9	Simpsons Gap, Northern Territory		
*gossei*	18049 (Latz 26971; NT D0209263)	24, 30, 53; 133, 25, 36	Henbury Homestead, Northern Territory	3.6	
*gossei*	16107	25, 21, 4; 131, 1, 31	Uluru, Northern Territory	3.6	
*gossei* †	17031	25, 21, 4.3; 131, 1, 32.2	Uluru, Northern Territory		36 here; 36, Tatemichi (1990); Kitamura *et al.* (2005)
*heterantha*	68204	20, 56, 41; 117, 36, 51	Mitchell River Bridge, Western Australia		
*heterantha*	16172	28, 59, 30; 121, 30, 15	Kookynie–Malcolm Road, Western Australia		
*heterantha*	68243	21, 50, 28; 114, 9, 16	Exmouth, Western Australia		
*heterantha* †	68249	23, 32, 23; 113, 57, 49	Minilya–Exmouth Road, Lyndon River crossing	2.5	48
*heterantha* †	68182	22, 21, 59; 119, 0, 19	Fortescue Marsh, Marillana Station, Western Australia	2.6	
*heterantha* †	68168	22, 26,24; 119, 40, 13	Fortescue Marsh, Roy Hill Station, Western Australia	2.5	
*ingulba*	18056 (Albrecht 13274; NT D0196653)	21, 39, 2; 134, 17, 9	Alyawarra Land Trust, Queensland		
*ingulba*	17027	25, 1, 25; 129, 25, 42	Lasseter’s Cave, Northern Territory	3.1	
*ingulba*	16085	24, 15, 16; 131, 30, 33	Watarrka National Park, Northern Territory		
*ingulba*	16179	28, 0, 30; 119, 19, 10	Sandstone, Western Australia	2.9	
*ingulba*	18010 (Gibson 6574; PERTH 8819629)	25, 18, 52.16; 120, 53, 23.91	Little Sandy Desert, Western Australia	3.8	
*ingulba* †	18059 (Latz 21389; NT A0110132)	23, 45, 6; 138, 24, 52	Ethabuka Homestead, Queensland		40 here; 40, Tatemichi (1990)
*ingulba* †	16095	25, 31, 8; 131, 48, 55	Mulga Park Road, near Mt Conner, Northern Territory	2.9	
*insecticida*	16038	23, 35, 30; 134, 20, 22	Mt Benstead Creek, Northern Territory		
*insecticida*	68228	22, 46, 50; 115, 4, 58	South-west of Nanutarra Roadhouse, Western Australia	3.1	
*insecticida*	68258	24, 45, 21; 114, 8, 10	Gascoyne River, Rocky Pool, Western Australia		
*insecticida*	18012 (Latz 955; CANB 207118.1)	25, 1, 54.48; 129, 27, 5.04	Tjukaruru Highway, Northern Territory		
*insecticida*	18026 (Latz 30922; NT D0273534)	22, 51, 40; 134, 27, 1	Alcoota Fossil Reserve, Northern Territory	2.9	42
*insecticida* †	68193	21, 15, 52; 117, 4, 25	Galah Siding, Millstream-Chichester National Park, Western Australia		42
*insecticida* †	68165	22, 37, 2; 119, 57, 36	Roy Hill Station, Western Australia		42
*karijini*	18009 (Anderson 172; PERTH 8437386)	22, 42, 4.7; 117, 23, 59	Mt Turner, Western Australia	3.3	40
*karijini*	68178	22, 23, 25; 118, 16, 3	Joffre Gorge, Karijini National Park, Western Australia	3.3	
*karijini*	18002 (Naaykens 15-5-J280; PERTH 8757682)	23, 15, 56; 117, 44, 18.7	Rocklea, Western Australia		
*maritima*	16119	34, 59, 29; 137, 45, 30	Wool Bay, South Australia		
*maritima*	16118	34, 49, 35; 137, 49, 29	St. Vincent Bay, South Australia		32 here; 32, Tatemichi (1990)
*maritima* †	Conran 3368 (AD)		York Peninsula, South Australia	3.4	
*maritima* †	18024 (Jones & Duval 140, AD 187318)	35, 37, 44; 138, 28, 28	Fleurieu Peninsula, Newland Head Conservation Park, South Australia		30
*megalosiphon*	17005	31, 3, 16.5; 147, 56, 3.6	Macquarie Marshes, New South Wales		40, Tatemichi (1990)
*megalosiphon*	17009	29, 34, 5.2; 149, 24, 22.9	Mehi River Bridge, New South Wales		
*megalosiphon*	18175	20, 37, 42; 143, 4, 4	Richmond, Queensland		
*megalosiphon* †	18167	22, 30, 24; 143, 22, 8	Landsborough Hwy (A2), approx. 38 km east of Longreach, Queensland	2.8	
*monoschizocarpa*	16004	14, 4, 12; 131, 15, 2	Oolloo Crossing, Northern Territory	4.3	
*monoschizocarpa*	16013	14, 54, 40; 133, 5, 12	Mataranka, Northern Territory		
*monoschizocarpa*	16010	14, 55, 17; 133, 8, 3	Waterhouse River, Northern Territory		
*monoschizocarpa* †	16005	14, 21, 48; 131, 33, 27	Claravale, Dorisvale Rd, Northern Territory	4.3	48 here; *n* = 24, gametic count, Horton (1981)
*murchisonica*	68279	27, 49, 40; 114, 41, 19	Murchison River Bridge, Western Australia		42
*notha*	17012	31, 54, 8.3; 150, 47, 11.4	Washpools Campground, New South wales	6.5	64
*paulineana*	Conran 3610 (AD)	32, 49, 55; 137, 7, 31	Corunna Station, South Australia	3.4	
*paulineana*	Conran 3353 (AD)	32, 49, 55; 137, 7, 31	Corunna Station, South Australia		
*paulineana*	16121	33, 4, 22; 138, 2, 33	Telowie Beach track, South Australia		32
*paulineana*	Conran 3617 (AD)	32, 41, 53; 137, 45, 8	Blanche Harbor, South Australia	3.4	
*paulineana* †	Conran 3612 (AD)	32, 40, 16; 137, 06, 50.2	Hills above Corunna Station, South Australia	3.2	
*sp. nov.* Burkett	68250	23, 32, 23; 113, 57, 49	Lake Macleod, Western Australia		
*sp. nov.* Burkett	68229	22, 41, 25; 114, 17, 58	Burkett Road, Western Australia	3.0	42
*sp. nov.* Burkett†	68270	25, 56, 44; 114, 18, 37	Junction Gladstone Road and Northwest Coastal Hwy, Western Australia	3.0	
*sp. nov.* Coondiner	68166	22, 26, 37; 119, 46, 45	Roy Hill Station, Northern Territory		
*sp. nov.* Coondiner	68161	22, 41, 54; 119, 44, 26	Coondiner Pool, Northern Territory	2.6	40
*sp. nov.* Hamelin	68277	27, 8, 46; 114, 37, 11	Nerren Nerren, Western Australia	3.2	42
*sp. nov.* Hamelin	68273	26, 24, 11; 114, 9, 59	Hamelin Pool, Western Australia		
*sp. nov.* Karara	68280	29, 11, 42; 116, 23, 16	Karara Mine, Western Australia	2.6	
*sp. nov.* Karara	68288	29, 20, 22; 116, 10, 2	Bowgada–Mullewa Road, Western Australia		
*sp. nov.* Karara†	16199	28, 36, 40; 116, 53, 44	Yalgoo–Paynes Find Road, north-west of Payne’s Find, Western Australia	2.7	
*sp. nov.* Kumarina	68157	25, 5, 42; 119, 22, 46	Kumarina, Western Australia	2.7	40
*sp. nov.* Maralinga	16142	30, 17, 59; 131, 36, 16	Maralinga, South Australia		
*sp. nov.* Maralinga	16143	30, 19, 47; 131, 35, 42	Maralinga, South Australia	3.4	
*sp. nov.* Simpsons	16049	23, 40, 37; 133, 43, 9	Simpsons Gap, Northern Territory	2.7	40
*sp. nov.* Strzelecki	18001 (Bates 84124; AD 239107)	28, 57, 50, 140, 7, 9	Strzelecki Track, South Australia	3.2	32
*sp. nov.* Strzelecki	17002	32, 17, 49.46; 142, 22, 6.68	Lake Menindee, New South Wales	3.2	
*sp. nov.* WAust	17016	34, 6, 16.9; 146, 11, 52.4	Whitton Stock Route, New South Wales	3.8	
*sp. nov.* WAust	17019	34, 4, 57; 146, 12, 58.3	Cocoparra, New South Wales	3.4	
*sp. nov.* WAust	18060 (Purdie 7721, CANB 789872.1)	32, 42, 17, 145, 38, 10	Yathong Nature Reserve, New South Wales		
*sp. nov.* WAust	18023 (Walsh 8382; MEL 2396268A)	38, 28, 40; 144, 63, 16	Mornington Peninsula, Victoria		30
*sp. nov.* Wongan	16207	30, 52, 29; 116, 45, 11	Wongan Hills, Western Australia		
*sp. nov.* Wongan	16204	30, 17, 7; 116, 39, 19	Dalwallinu Bushland Reserve, Western Australia		40
*sp. nov.* Wongan†	16208	30, 51, 44; 116, 37, 15	Wilding Road, Wongan Hills, Western Australia		44
*obliqua*	16096	25, 22, 51; 131, 50, 55	Mulga Park Road, Northern Territory		
*obliqua*	16141	30, 8, 46; 131, 30, 22	Maralinga, South Australia		
*obliqua*	Conran 3615 (AD)	32, 40, 8.2; 137, 8, 0.5	Corunna Station, South Australia		
*occidentalis*	68216	20, 18, 34; 118, 35, 3	Port Hedland, Western Australia		42, Tatemichi (1990)
*occidentalis*	68234	22, 25, 51; 114, 1, 31	South of Exmouth, Western Australia		
*occidentalis*	68202	20, 37, 34; 117, 11, 49	Point Samson, Western Australia	2.9	
*occidentalis* †	68205	20, 56, 41; 117, 36, 51	North-west Coastal Highway, bridge over Sherlock River, Western Australia	3.0	
*rosulata*	16188	28, 9, 15; 117, 41, 17	Mt Magnet, Western Australia	2.8	
*rosulata*	16170	28, 55, 2; 121, 28, 53	Leonora, Western Australia	2.7	40 here; 40, Tatemichi (1990)
*rosulata*	68264	25, 17, 33; 115, 42, 9	West of Gascoyne Junction, Western Australia		
*rotundifolia*	16161	31, 10, 51; 120, 23, 8	Boondi Rock, Western Australia	2.6	
*rotundifolia*	16157	32, 23, 42; 121, 46, 12	Dundas Rock, Western Australia		
*rotundifolia*	18051 (Gibson & Langley 5297; CANB 819368.1)	30, 26, 5; 120, 39, 11	Coolgardie North Road, Western Australia	2.6	
*rotundifolia*	18035 (Hislop *et al*., 173-37; PERTH 7433123)	32, 0, 37; 117, 22, 13	Quairading Community Bushland Reserve, Western Australia		42 here; 44, Tatemichi (1990)
*salina*	68283	29, 11, 13; 116, 27, 41	Weelhamby Lake, Western Australia	3.2	42 here; 42, Tatemichi (1990)
*sessilifolia*	16027	23, 59, 23; 133, 26, 9	Lawrence Gorge, Northern Territory		
*sessilifolia*	16069	23, 41, 11; 132, 40, 27	Glen Helen, Northern Territory		
*sessilifolia*	16016	22, 7, 54, 133, 24, 15	Ti-Tree, Northern Territory		
*sessilifolia*	18191	20, 40, 57; 139, 29, 45	Leichhardt River crossing, Northern Territory		
*sessilifolia* †	16215	23, 44, 48; 134, 0, 54	Jessie Gap, Northern Territory		40
*sessilifolia* †	18188	19, 49, 33; 140, 9, 9	Kajibbi/Kamilaroi Road, near Coolullah Station, Queensland	2.9	
*sessilifolia* †	18189	20, 26, 24; 140, 19, 11	Burke Developemental Road, Corella River bridge, north-west of Cloncurry, Queensland	2.9	
*simulans*	Conran 3560 (AD)	29, 7, 57; 134, 34, 21	West of Coober Pedy, South Australia	2.9	42 here; 40, Tatemichi (1990)
*simulans*	Conran 3559 (AD)	29, 14, 13; 134, 42, 54	South of Coober Pedy, South Australia		
*simulans*	16092	25, 10, 39; 133, 24, 1	Idracowra Station, Northern Territory		
*simulans* †	18055 (Schubert 572, NT D0269780)	29, 27, 24; 133, 6, 32	Tallaringa Conservation Reserve, South Australia	2.8	
*stenocarpa*	16167	29, 18, 11; 121, 29, 5	Koolkynie–Leonora Road, Western Australia		40
*stenocarpa*	16190	28, 10, 33; 117, 25, 29	Mt Magnet, Western Australia		
*stenocarpa*	16181	28, 0, 39; 118, 40, 24	Sandstone, Western Australia	2.9	
*stenocarpa* †	16176	28, 8, 59; 120, 34, 9	Leonora–Agnew Road, Western Australia	2.7	
*suaveolens*	17022	37, 29, 27; 148, 10, 7.6	Buchan, Victoria		32 here; 32, Tatemichi (1990); Kitamura *et al.*, (2005)
*suaveolens*	17021	36, 53, 17.5; 148, 25, 14.5	Snowy River, New South Wales	3.5	
*suaveolens*	17014	33, 49, 13.07; 150, 1, 34.4	Jenolan Caves, New South Wales		
*suaveolens*	17035	34, 18, 48; 149, 57, 53	Wombeyan Caves, New South Wales	3.3	
*truncata*	Conran 3599 (AD)	28, 1, 29.3; 135, 6, 6	Aloorina Creek, South Australia		36, Symon (1998)
*truncata*	Conran 3562 (AD)	28, 49, 8; 135, 1, 57	Moon Plain, South Australia	3.9	
*umbratica*	68208	21, 36, 15; 119, 1, 38	Woodstock–Marble Bar Road, Western Australia		46, Tatemichi (1990); Kitamura *et al.* (2005)
*umbratica*	68211	21, 30, 51; 119, 24, 58	Shaw River crossing, Western Australia	3.6	
*umbratica*	68214	20, 26, 51; 119, 59, 30	Shay Gap Road, Western Australia	3.8	
*velutina*	16035	23, 44, 48; 134, 0, 54	Jessie Gap, Northern Territory		
*velutina*	18037 (Pennay 749; BRI AQ826759)	25, 52, 10, 138, 35, 41	Munga-Thirri, Queensland		
*velutina*	Conran 3585 (AD)	28, 1, 52; 135, 54, 45	William Creek–Oodnadatta Road, South Australia		32 here; 32, Tatemichi (1990)
*velutina*	17003	30, 32, 5.99; 145, 6, 51.92	Louth, New South Wales		
*velutina*	18018 (Jeanes 2482; MEL 2338142)	34, 39, 13; 141, 48, 0	Murray-Sunset National Park, Victoria	3.3	
*velutina* †	16131	32, 37, 5; 135, 15, 40	Minnipa–Yardea Road, South Australia	3.3	
*velutina* †	18061B	23, 44, 20; 133, 57, 4	Emily Gap, Northern Territory	3.2	
*velutina* †	18066 (Kemp 11699; BRI AQ0797715)	23, 49, 50; 138, 30, 55	Ethabuka Station, Queensland		32
*walpa*	16116	25, 17, 6; 130, 43, 36	Valley of the Winds, Northern Territory	2.7	40
*walpa*	16105	25, 17, 12; 130, 44, 53	Valley of the Winds, Northern Territory	2.7	
*walpa*	16056	23, 49, 3; 132, 18, 57	Namatjira Drive, Northern Territory		
*yandinga*	16125	33, 24, 46; 136, 16, 23	Carappee Hill, South Australia		
*yandinga*	16135	33, 9, 36; 134, 39, 32	Venus Bay, South Australia		
*yandinga*	Conran 3853 (AD)	32, 58, 16; 135, 33, 57	Wudinna Hill, South Australia		
*yandinga*	16129	34, 38, 21; 135, 21, 10	Coffin Bay, South Australia	3.3	
*yandinga*	16126	34, 49, 49; 135, 46, 52	Sleaford Mere, South Australia		
*yandinga* †	16134	32, 33, 27; 135, 19, 17	Yandinga Gorge, Gawler Ranges, South Australia		30

For the chromosome numbers, we also report published counts from the literature.

^*^Chase and Christenhusz, unless otherwise noted. For accessions retrieved via seeds removed from herbarium specimens, the Chase and Christenhusz numbers are provided for the voucher prepared from the cultivated material; the collector and number for the original herbarium specimen are also provided (including the herbarium accession number).

†Accessions not in the RADseq tree.

We made efforts to include the same accessions that we studied for chromosome numbers and genome sizes in the phylogenetic analysis, but this was not always possible. Chromosome numbers in most species were studied either to verify previous counts from the literature or to re-confirm our first counts (if they deviated from those in the literature), but in a few cases we have relied solely upon counts from the literature (i.e. *N. rosulata* and *N. truncata*). In only a few cases did we find something that contradicted what had been published previously, and fortunately genome sizes did not vary enough to make the few unsampled accessions problematic for an examination of general trends in genome sizes vs. chromosome numbers. We have not included parental diploids in this phylogenetic study because the most recent common ancestor of *Nicotiana* sect. *Suaveolentes* and any diploid relatives is millions of years greater than the age of the target group (Clarkson *et al.*, 2017). Combining diploids and allotetraploids in the same phylogenetic analysis could also be highly problematic due to the difficulties of confusing maternal and paternal copies, so we confined our phylogenetic studies to just the species of *Nicotiana* sect. *Suaveolentes,* minimizing paralogy issues (Brandrud *et al.*, 2020).

Given the phenotypic plasticity in the group and the number of revised species concepts and new species that we have identified, we consider our species determinations more reliable for counts that deviate from those in the literature. We are in the process of identifying vouchers made for the older studies, but many of these were never clearly marked as such in Australian herbaria. In some cases, vouchers were never made. As far as possible, we have included an accession from the same locality as the specimen designated as nomenclatural type, e.g. if we have distantly related genetic clusters of accessions that have been identified previously as in *N. rosulata*, we have designated as *N. rosulata* the cluster with the accession from the type locality (e.g. in the case of *N. rosulata*, the type was collected near Leonora, Western Australia, so the accessions that cluster with the material collected in Leonora are labelled as *N. rosulata*). For those species in which we report infra-specific chromosome variation, e.g. *N. goodspeedii* and *N. benthamiana*, the exact accessions used in the cytological and genome size studies are included in the RADseq matrix (Table 1).

### Collecting and import permits

All field-collected material is covered under the following collecting permits: Western Australia SW017148, CE006044, Northern Territory 58658, Victoria 10008399, New South Wales SL101924 and Queensland PTU-18001061. Permission to remove seeds from herbarium specimens was obtained from the curators/collection managers of the following herbaria: BRI, NT and PERTH. All seeds were imported into the UK following published guidelines, and plants were grown in quarantine at the Royal Botanic Gardens, Kew, UK import permit DEFRA PHL2149/194627/5NIRU CERT:106-2019; HMRC TARIFF CODE: 0601209090.

### DNA isolation and sequencing

Total DNA was isolated from silica-dried leaves using a cetyltrimethylammonium bromide (CTAB) procedure (Doyle, 1990), after a 20 min pre-treatment on ice with ice-cold sorbitol buffer (100 mm Tris–HCl, 5 mm EDTA, 0.35 m sorbitol, pH 8.0). After extraction, the DNA was further treated with 2.5 µL of RNase A (Thermo Fischer, USA) for 30 min at 37 °C and the reaction cleaned with a NucleoSpin gDNA clean-up Kit (Machery-Nagel, Germany), following the manufacturer’s instructions. DNA was quantified with a Qubit 3.0 spectrophotometer (Thermo Fisher Scientific, USA).

Single-digest RADseq libraries were prepared following a protocol successfully used in previous studies (e.g. Heckenhauer *et al.*, 2018; Brandrud *et al*., 2019, 2020). The protocol used the restriction enzyme PstI to treat batches of 60 individuals per library, including any necessary repeats when not enough had been initially obtained. The inline and index barcodes used differed from each other by at least three positions. The libraries have been sequenced at the Vienna BioCenter Core Facilities (VBCF; https://www.viennabiocenter.org/) on an Illumina Hiseq 2500 as pair-end reads of 125 bp.

### Bioinformatic and phylogenomic analyses

The raw reads were demultiplexed first based on index barcodes using BamIndexDecoder v.1.03 (included in the Picard Illumina2Bam package, available from http://gq1.github.io/illumina2bam/). Demutiplexing based on inline barcodes was then conducted with process_radtags from Stacks v.1.74 (Catchen *et al.*, 2013), together with quality filtering that removed reads containing any uncalled base and those with low quality scores, but rescued barcodes and cut sites with maximum one mismatch.

The reads were mapped with bwa mem (v.0.7.17-r1188; Li and Durbin, 2009) to a reference genome for a member of this section, *N. benthamiana* (v.1.0.1, Bombarely *et al.*, 2012), a species widely used as a model organism in plant virology and biotechnology (Tregoning *et al.*, 2020). Given that the parents of these allotetraploids were relatively distantly related to each other (from different taxonomic sections; Chase *et al.*, 2003; Clarkson *et al.*, 2004) and that extensive post-WGD chromosomal evolution has already taken place during diploidization, our approach in using a reference genome within the group circumvents as much as possible paralogy issues and can treat the data as effectively ‘diploid’ (i.e. the homoeologous sequences are expected to map to their own parental sequence). During mapping, the option –M was applied to flag shorter split hits as secondary. The individual mapping rates were investigated to test for mapping bias, potentially driven by phylogenetic relatedness to the reference individual. The resulting aligned sam file was sorted by reference coordinates, and read groups were added using Picard Toolkit v.2.18.17 (available from http://broadinstitute.github.io/picard/). Indel realignment was performed with the Genome Analysis Toolkit v.3.8 (McKenna *et al.*, 2010), thinning the data to a maximum of 100 000 reads per interval. A catalogue has been built and variants were called from the realigned .bam files with the ref_map.pl pipeline in Stacks with default settings. Export_sql.pl and populations from Stacks were used to extract those regions with up to 40 single nucleotide polymorophisms (SNPs) and which had data for at least 50 % of the individuals. We also retained only those variants with a maximum observed heterozygosity of 0.65 to avoid further use of any pooled paralogues. Final filtering of the SNPs was applied in vcftools v.0.1.13 (Danecek *et al.*, 2011) to remove indels and retain only SNPs with a minor allele frequency ≥0.014 (i.e. present in at least four haplotypes). The data were filtered for percentage missing in steps of 5 % from 0 to 20 %, and the optimum level was determined to maximize the average bootstrap support. The filtered. vcf files were transformed in PHYLIP format with PGDspider v.2.1.1.0 (Lischer and Excoffier, 2012) and invariant sites were removed with the script ascbias.py (https://github.com/btmartin721/raxml_ascbias).

Maximum likelihood (ML) trees were calculated with the software RAxML v.8.2.11 (Stamatakis, 2014). The analyses were performed with 1000 rapid bootstrap replicates, using an ascertainment bias correction to the likelihood calculations (Lewis, 2001) as recommended for concatenated SNPs. A simultaneous search for the best-scoring ML tree was conducted with a general time-reversible model of nucleotide substitutions (i.e. the GTRCAT model) and disabled rate heterogeneity among sites model (i.e. –v). The best tree was then visualized and annotated in R, using ape v.5.3 (Paradis and Schliep, 2018), biostrings (Pagès *et al.*, 2020), ggplot2 (Wickham, 2016), ggtree (Yu *et al.*, 2017) and treeio (Wang *et al.*, 2020). We assigned *N. africana* as the outgroup because it was well supported as sister to the rest of *Nicotiana* sect. *Suaveolentes* in several phylogenetic analyses using both plastid and nuclear data (Chase *et al.*, 2003; Clarkson *et al*., 2004, 2010, 2017; Marks *et al.*, 2011; Kelly *et al.*, 2013).

To assess patterns of hybridization/introgression, we constructed a co-ancestry heatmap (Fig. 2) for a set of 64 accessions, corresponding to the accessions in sub-tree B (Fig. 3). For this purpose, we used the genotype-free method implemented in ANGSD v.0.9.10 (Korneliussen *et al.*, 2014) on the indel-realigned .bam files to calculate genotype likelihoods as these were shown to be accurate estimates of genomic parameters for medium to low coverage data (Maas *et al.*, 2018; Warmuth and Ellegren, 2019). Only sites with data for at least 75 % of individuals were retained with a minimum 20 base quality and mapping quality. For 1 085 059 high-confidence (*P* < 1e-6) variable positions that had a minor allele shared by at least three individuals, we inferred the major and minor alleles frequencies under a GATK-based genotype likelihood model. Starting from covariance matrices calculated using pcangsd v.0.99 (Meisner and Albrechtsen, 2018) from the genotype likelihoods, we further visualized co-ancestry of the different accessions using the heatmaps.2 function (GPLOTS v.3.0.1.1; Warnes *et al.*, 2020).

### Chromosome number determination

We used the following protocol for determining chromosome numbers. We first re-potted mature but still actively growing plants in the greenhouse about 2 weeks before harvesting root tips. This forced the plants into producing many actively growing roots, increasing the number of root cells with acceptable mitotic figures. Young root tips were obtained directly from cultivated material and pre-treated with 0.002 m 8-hydroxyquinoline at 10–12 °C for 24 h. Subsequently, the roots were fixed in Farmer’s fixative (3:1 absolute ethanol:glacial acetic acid, v/v) for 2–24 h at room temperature. The roots were then washed twice in distilled water (10 min each or until they sank to the bottom of the tube). For slide preparation, the roots were digested on the slide with an enzymatic solution containing 2 % (w/v) cellulase and 20 % (w/v) pectinase in phosphate buffer at 37 °C for 2 h in a wet chamber and washed subsequently to remove the enzyme with a solution containing distilled water and glacial acetic acid (1:1, v/v) for 1 h in a wet chamber. After washing, the meristematic tissue was fragmented with needles in a drop of 45 % acetic acid, placed under a coverslip and squashed. The slide/coverslip assembly was frozen in liquid nitrogen for 5 min, and the coverslip was removed quickly with a razor blade and the slide air dried. Fluorochrome staining followed Schweizer (1976). The slides were first stained with chromomycin A3 (CMA; 0.2 mg mL^−1^) for 1 h and then with 4′,6-diamidino-2-phenylindole (DAPI; 2 μg mL^−1^) in water for 30 min before mounting in glycerol/McIlvaine buffer medium. The best cells were captured on a Zeiss light microscope using an Axio Cam MRC5 video camera and Axiovision 4.8 software. Chromosome images were processed in Photoshop CS3, and counts and measurements were obtained with the software ImageJ.

### Genome size estimation

Genome sizes were estimated using seeds instead of leaves or floral tissues. We originally worked with leaf tissue but found that this made genome size estimates difficult or impossible for some species for reasons that are unclear. Perhaps secondary chemistry or unusual leaf pigments negatively impacted the estimates, whereas we experienced few problems using seeds. The genome sizes of these *Nicotiana* species were measured using a modification of the approach detailed in Pellicer and Leitch (2014). Briefly, 5–10 *Nicotiana* seeds were co-chopped with a razor blade and 2 cm^2^ of leaf from the size standard, *Petroselinum crispum* (1C = 2.22 Gb/1C; Apiaceae) in 2 mL of isolation buffer (general purpose buffer of Loureiro *et al.*, 2007) supplemented with 0.3 % polyvinylpirrolidone (PVP-40, Sigma Aldrich) and 0.04 % β-mercaptoethanol. The chopped material was then filtered through a 30 μm nylon mesh, stained with propidium iodide (1 mg mL^–1^; Sigma Aldrich in water) at a final concentration of 50 μg mL^–1^, and stored on ice for 10–40 min. Three replicate runs per species were conducted, recording 5000 particles using a Partec Cyflow Space with a 532 nm (Partec GmbH, Münster, Germany) flow cytometer fitted with a green laser (30–100 mW). FLOWMAX software (v. 2.7; Partec GmbH). We included here none of the previous estimates (ten in Narayan, 1987) because there were no vouchers made (the seeds were taken from seed banks, which contain no specific information on provenance) and thus we could not be certain about which species were analysed.

### Analyses of genome size and chromosome number change

BayesTraits v.3.0.2 (Pagel *et al.*, 2004) was used to infer genome size change across the tree. We used ChromEvol v.2.0 (Glick and Mayrose, 2014) for the analysis of chromosome number change. A suitable tree for modelling both phenomena was created by pruning the tree in Fig. 3, leaving only one representative of each taxon. In taxa with three or more accessions, the accession with the median branch length was chosen as the representative. Tree editing was done in R using ape (Paradis and Schliep, 2018).

Genome size change in *Nicotiana* sect *Suaveolentes* was estimated using the continuous model in BayesTraits, with the tree branches scaled to 0.01, and estimating the delta, kappa and lambda parameters. The Markov chain had 11 000 000 iterations, sampled every 500 iterations with a burn-in of 1 000 000 iterations. Estimation of ancestral genome size was limited to values between 2.5 and 6, the range we observed in these species.

The ancestral chromosome number was assessed using the default models, with the root node fixed to *n* = 24 based on the number of chromosomes in the diploid parents (*n* = 12). Constant rate models were equal and performed better than linear dependence models. The simplest model was chosen, assessing chromosome gains and losses (all species have the same ploidy, so duplications were not investigated). Another model was constructed in which no chromosome number increases were allowed, again with the root node fixed to be *n* = 24. All models were run with 100 000 simulations of changes along the branches. Our favoured scenario does not permit number increases, and our assumptions for this are presented in the Discussion, but this choice of model does not affect our general conclusions. Results were checked in Tracer v.1.6 and visualized in R with the packages ape (Paradis and Schliep, 2018), treeio (Wang *et al.*, 2020), gtree (Yu *et al.*, 2017), patchwork (Pedersen, 2020) and ggimage (Yu, 2020).

A simple linear (Brownian motion) model was fitted to test for a specific association between genome size and chromosome number (all included species are of the same ploidy), with the former as the dependent variable and the latter as the independent variable. To account for evolutionary non-independence between taxa, we also estimated phylogenetic independent contrasts (PICs) for genome size and chromosome number based on the phylogenetic tree in Fig. 4, using ape in R (Paradis and Schliep, 2018). The tree was pruned to include one representative of each taxon for which both data types were available. The PICs were regressed through the origin (Garland *et al.*, 1992) to test whether there was a linear relationship between these two. If there are only two variables, in this case genome size and chromosome number, PIC is equivalent to using the phylogenetic generalized least squares procedures (PGLS; Blomberg *et al.*, 2012). The difference between PIC and PGLS is that the latter returns an intercept, but the slope parameter (which represents the relationship between genome size and chromosome number) is identical.

## RESULTS

### Phylogenetic analyses

After demultiplexing and quality filtering, we retained on average 2.0 million pairs of reads per accession (s.d. 0.6 million). The data have been deposited in the NCBI Sequence Read Archive (BioProject ID PRJNA681916, SRA Study SRP295424). Mapping success to the reference of *N. benthamiana* was very high, with an average 96.1 % (s.d. 4.8 %). No phylogenetic mapping bias could be observed (e.g. mapping rates for the two accessions of *N. africana*, the outgroup, were 96.5 % and 97.1 %). The average coverage across samples obtained after mapping was 11.1× (s.d. 2.2×).

After filtering, numbers of retained SNPs ranged between 130 995 (with data for at least 95 % individuals) and 599 473 (with data for a minimum of 80 % individuals). After comparing the average bootstrap support, the dataset including up to 15 % missing data (i.e. including 457 382 SNPs) was chosen as the final matrix.

The ML tree produced (Fig. 3A, B) exhibits well-supported interspecific relationships [bootstrap percentage (BP) 100], and multiple accessions of species form unique, well-supported clusters. The only major lack of resolution is close to the base, where the position of *N. forsteri* relative to *N. monoschizocarpa* is not well supported (BP 73). The 18 major clades identified (numbered as Roman numerals, I–XVIII) are each generally widespread geographically and occur in a variety of habitat types, varying from sheltered (i.e. under trees such as mulga, *Acacia aneura*, or on the south sides of rock outcrops and in gorges) to open (i.e. sand dunes, dry riverbeds, fields, ruderal sites and gibber plains). The newly recognized species (Chase and Christenhusz, 2018*a*, *b*, *c*, 2021*a*, *b*; Chase *et al*., 2018*b*, 2018*c*, 2021*b*, *c*, *d*) are clearly distinct from the concepts in which they were previously included. For example, *N. karijini*, for which herbarium specimens had been identified previously as *N. umbratica*, is sister to *N. benthamiana* (clade VII); *N. gascoynica*, previously considered to be specimens of *N. simulans*, is sister to the *N. simulans* clade (clade XI) plus *N. cavicola* (clade X); *N. yandinga*, previously identified as *N. maritima*, is sister to the whole of the *N. suaveolens* clade (clade XVIII, which includes *N. maritima*); and, finally, *N. faucicola*, which had also routinely been identified as *N. maritima* (and occasionally as *N. velutina*; clade XVII) is sister to *N. suaveolens* plus another as yet undescribed species in the larger *N. suaveolens* clade (clade XVIII).

The major clades identified in the RADseq tree largely conform to the distribution of major differences in vestiture observed, e.g. clade XI with sparse, long, multicellular straight gland-tipped hairs, clade XIII with dense long and short gland-tipped hairs, and clade XVIII with long, curly (wooly), multicellular, gland-tipped hairs, but few other major morphological characteristics seem to co-vary with the genetic results for the larger multispecies clades. We are investigating seed morphology, which is variable and potentially taxonomically useful.

Generally, the Australian species of *Nicotiana* are morphologically similar and not easily distinguished, especially if one is working with the fragmentary material typical of many herbarium specimens. Inflorescence structure and vestiture are useful traits, with floral traits, especially size, useful in some cases for distinguishing closely related species from each other. Despite their overall highly similar morphology/habit, the high levels of bootstrap support make this a good phylogenetic framework for examining how chromosome number and genome size vary. Some hybrids have been detected, including one that is a neo-allotetraploid (*N. notha*; Figs 2 and 3B), but all other obvious hybrids have been excluded from this study. As for *N. notha*, in our results hybrids are obvious due to their isolated positions as sister accessions to larger clades and clear genetic similarities to at least two other species in heatmaps. In Fig. 2, *N. notha* displays general genetic similarities (brighter colour) to two of the larger clades, XVII and XVIII (Fig. 3B), but specifically *N. sp. nov*. Strzelecki and *N. sp. nov.* WAust (bright rose in Fig. 2), which both occur in the general area in which this material was collected (Table 1). Both putative parents are *n* = 16 and have genome sizes estimated at 3.2 and 3.4–3.8 pg, respectively (Table 1), and *N. notha* has *n* = 32 and 6.5 pg. We have found herbarium specimens of this same entity in other nearby localities (labelled as *N. suaveolens*), so this allotetraploid clearly occurs in more than one place and putatively functions as a species, warranting its formal description (Chase *et al.*, 2021*d*).

### Chromosome number change

Chromosome numbers are shown on the summary RADseq tree (Fig. 4B, C), the data being a combination of our own counts and those taken from the literature (Table 1); however, in the few cases for which our counts differ from earlier reports, we show only our results because we are not sure of the species determinations of previous researchers (and we have been unable to examine the vouchers). Species varied in chromosome number, with numbers forming an almost complete descending dysploid series, ranging from *n* = 24 in *N. monoschizocarpa* and *N. heterantha* to *n* = 15 in *N. yandinga*, *N. maritima*, *N. faucicola* and *N. suaveolens*. Chromosome morphology is also highly variable among species, with the occurrence of metacentric, sub-metacentric and acrocentric chromosomes (F. Nollet and M. W. Chase, unpubl. res.), but these are not presented because they are not a focus of this study.

Intraspecific chromosome number variation was observed in two species: *N. benthamiana* with *n* = 18, 19; and *N. goodspeedii* with 2*n* = 20, 21. Chromosome number variation appears in some cases consistent within the major clades, e.g. *n* = 21 in clade XIII, *n* = 20 in clade XI, *n* = 16, 18 in clade XVII and *n* = 15, 16 in clade XVIII; however, in other clades, numbers vary considerably. The species exhibiting the ancestral or near ancestral chromosome number, *n* = 23, 24 (the diploids hypothesized to be the parents of these allotetraploid species are both *n* = 12; Chase *et al.*, 2003) – *N. africana*, *N. forsteri*, *N. monoschizocarpa*, *N. heterantha* and *N. umbratica* (clades I–VI) – are all located on the basal nodes of the tree, with these nodes reconstructed as *n* = 24 (Fig. 4C). Lower chromosome numbers are found independently in four clades, VII (*n* = 18–20), XIV (*n* = 18, 19), XVII (*n* = 16–18) and XVIII (*n* = 15–20).

Under our favoured scenario (see the Discussion) in which increases in chromosome number are not permitted (Fig. 4C), the spine of the tree exhibits a stepped decrease at each node in which *n* = 24–22 occur in sequence, with subsequent multiple independent decreases within many clades. Near the tips of the tree, changes in the spine are precipitous, e.g. skipping from *n* = 21 to 18 and 16 in clades XVII and XVIII, respectively (Fig. 4C). If a model is applied in which increases and decreases are equally likely, then there is no clear pattern of chromosome number change along the spine of the tree (Supplementary data Fig. S1), but rather it is focused largely within the major clades, resulting in both decreases and increases. For example, *N. goodspeedii* (*n* = 20, 21) is surrounded by species with lower numbers (*n* = 15–18), so under this model an increase is hypothesized in *N. goodspeedii*. In our favoured model (Fig. 4C), the spine node for this group is *n* = 21, so changes are all decreases in chromosome number. Our choice of model does not affect our general conclusions about the interactions between genome size and chromosome number change.

### Genome size change

Genome size for the allotetraploid ancestor of *Nicotiana* sect. *Suaveolentes*, which was hypothesized as *n* = 24, could be expected to be in the range of 4.8–5.2 pg per 1C nucleus (see the Discussion), corresponding roughly to that of *N. africana* (*n* =23) with 5.4–5.5 pg/1C. A decidedly smaller genome size was recorded in two of the *n* = 24 species, *N. monoschizocarpa* with 4.3 pg/1C and especially *N. heterantha* with 2.5 pg/1C. No chromosome or genome size data are available for *N. fatuhivensis* due to lack of access to appropriate material.

After the above species diverged, genome size (Fig. 4A, B) is estimated to become uniform along the spine of the tree, 3.2–3.3 pg/1C, as well in clades VII (3.3–3.4 pg/1C) and VIII (3.2 pg/1C), then dropping slightly in clades IX (2.7 pg/1C), X (2.7 pg/1C), XI (2.7–2.9 pg/1C) and XII/XIII (2.9 pg/1C). In clade XIV (*n* = 18, 19) and its sister clade XV (*n* = 20, 21), genome size ranges from 3.4 to 3.9 pg/1C and from 2.6 to 3.0 pg/1C, respectively. Finally, in the clades with the lowest chromosome numbers, XVII and XVIII, genome size is uniformly 3.2–3.6 pg/1C and close to the estimated ancestral genome size of the core group of species and along the spine (3.2–3.3 pg/1C). Thus, it appears that chromosome numbers and genome sizes are not co-varying (Fig. 4B); one species with the ancestral number, *N. heterantha* with *n* = 24, has among the lowest genome sizes (2.5 pg) in the group, and those with the lowest chromosome number, *n* = 15, 16 (clades XVII and XVIII) are uniformly larger (some up to 30 %) than those in several clades with *n* = 20, 21. We have left the neo-allotetraploid, *N. notha*, *n* = 32 and genome size of 6.5 pg/1C, out of these comparisons. Notably, as chromosome numbers decrease in clades XIV, XVII and XVIII (*n* = 15–19), genome size appears to stabilize or even increase relative to that estimated along the spine (Fig. 4A, B).

**Fig. 5.**
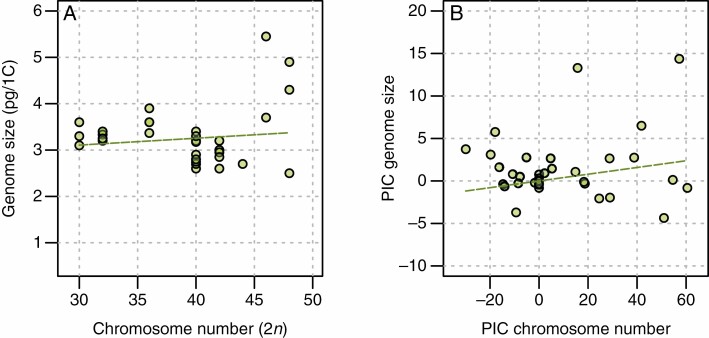
Scatter plots showing the relationships between (A) genome size and chromosome number; and (B) genome size and chromosome number using phylogenetic independent contrasts (PICs). The dashed trend line indicates the estimated slope from a linear regression, which is not significant in either (A) or (B).

No association was found between genome size and chromosome number [B = 0.0148, confidence interval (CI) = –0.0262 to 0.0558, *P* = 0.4681, adjusted *R*^2^ = –0.013; Fig. 5A; Table 2]. Similarly, using PICs did not show a significant relationship between genome size and chromosome number (B = 0.0396, CI = –0.0137 to 0.0930, *P* = 0.14, adjusted *R*^2^ = 0.0342; Fig. 5B; Table 2).

**Table 2.** Output from simple linear models

(A)	Genome size, approximate chromosome number						
	Adjusted *R*^2^ = –0.01299						
		Coefficient	Lower CI (2.5 %)	Upper CI (97.5 %)	s.e.	*t*-value	*P*-value
	Intercept	2.6624	1.0473	4.2775	0.7956	3.3470	0.00196
	CN	0.0148	–0.0262	0.0558	0.0202	0.7340	0.46806
(B)	PICs GS ~ PIC CN						
	Adjusted *R*^2^ = 0.03419						
		Coefficient	Lower CI (2.5 %)	Upper CI (97.5 %)	s.e.	*t*-value	*P*-value
	picCN	0.0396	–0.0137	0.0930	0.0263	1.5080	0.1400

Output testing the association between (A) genome size (GS) and chromosome number (CN); and (B) GS and CN with phylogenetic independent contrasts (PICs).

### Data availability

The data underlying this article are available in the NCBI Short Reads Archive and can be accessed with BioProject ID PRJNA681916, SRA Study SRP295424.

## DISCUSSION

### Chromosome number change and environmental correlates

Post-WGD, the general pattern of chromosome number change is reduction during diploidisation (Wendel, 2015; Dodsworth *et al*., 2015; Soltis *et al.*, 2016; Escudero and Wendel, 2020), which is most obvious in herbaceous groups, as observed here in *Nicotiana* sect. *Suaveolentes*, or those with herbaceous ancestry, such as the now mostly woody families in Malpighiales (e.g. Passifloraceae) and Lamiales (e.g. Oleaceae, Bignoniaceae), among others (Carlquist, 2009; chromosome data from the *Index to plant chromosome numbers*; Goldblatt and Johnson, 1979–onwards).

The general background for chromosome number change in *N.* sect. *Suaveolentes* is one in which the species with higher numbers occur in the more dependably wet habitats in northern (summer monsoon) and eastern (rain forest) Australia. This group began to radiate in the arid zone only within the last 2 million years (Clarkson *et al.*, 2017; Cauz-Santos *et al*., 2022), with formation of the core group of species (clades VII–XVIII), in which chromosome number and genome size both exhibit decreases in general. In all species with <20 pairs of chromosomes (four independent cases), genome size stops decreasing or even increases by up to 30 % compared with relatives with ≥20 pairs (Fig. 4; see below). We appear to have detected a chromosome number inflection point at which genome size begins to stabilize or even increase (see below).

Carta *et al.* (2018) showed in a phylogenetic context for Italian endemic plants that open, disturbed, drought-prone habitats select for low chromosome numbers, whereas long-lived species occurring in shaded, stable habitats are associated with higher chromosome numbers. Similarly, those *Nicotiana* species with the lowest chromosome numbers (*n* = 15, 16) occur in the uniformly driest regions in southern Australia (many with <200 mm of rain per year) and those with the higher numbers in the wetter parts of northern and eastern Australia. These observations support the hypotheses of Darlington (1937) and Stebbins (1950) that environmental instability and stress favour the lower levels of recombination brought about by fewer chromosomes. Protection from interspecific gene flow and recombination of adapted, linked alleles may therefore be the most important effects of changes in chromosome structure (Rieseberg, 2001). The radiation of these *Nicotiana* species occurred against a background of diploidization associated with invasion of novel habitats, a phenomenon compatible with the lineage-specific ohnologue resolution (LORe) model (Robertson *et al.*, 2017). The redundant, modular structure of duplicated gene regulatory networks offers all polyploid species increased possibilities for novel evolutionary innovation and adaptation through mutations. Whether the radiation of the species of *N*. section *Suaveolentes* in the Australian arid zone is specifically adaptive has yet to be documented. Furthermore, there are no explanations for why lower chromosome numbers are so routinely associated in angiosperms with the evolution of annual life histories and inbreeding from outcrossing perennial ancestors (as in these species of *Nicotiana*).

### Drivers of genome size vs. chromosome number change

The ancestral chromosome number in *N.* sect. *Suaveolentes* should be *n* = 24 (the sum of those in the putative parents, *n* = 12; Chase *et al.*, 2003), which is found in species at the first several nodes in the RADseq tree (Fig. 4B, C). Based on the genome size of *N. sylvestris*, 2.70 pg/1C (closely related to the paternal diploid parent; Leitch *et al.*, 2008; Clarkson *et al.*, 2010), *N.* sect. *Noctiflorae*, 4.18 pg/1C (Clarkson *et al.*, 2004; Kitamura *et al.*, 2005; Kelly *et al.*, 2013), and *N.* sect. *Alatae*, *n* = 3.7 pg/1C (Chase *et al.*, 2003; Kitamura *et al.*, 2005), we estimate that the ancestral genome size of *N.* sect. *Suaveolentes* might be in the 5.40–6.88 pg/1C range. The genome size of *N. africana* (*n* = 23), which is sister to the rest of *N.* sect. *Suaveolentes*, is 5.45 pg, and is thus close to the ancestral size.

*Nicotiana forsteri* (*n* = 24) with 4.9 pg/1C (Table 1; Fig. 4B, C) is also close to the expected genome size range, whereas *N. monoschizocarpa* (*n* = 24) with 4.3 pg/1C and particularly *N. heterantha* (*n* = 24) with 2.5 pg/1C deviate strongly from the expected genome size and have clearly followed an independent path of reduction, with the last being among the smallest genomes in *N.* sect. *Suaveolentes*. The genome size of *N. heterantha* is also lower than those in the South American diploid progenitors of *N.* sect. *Suaveolentes* (see also below). Although *N. umbratica* (clade VI) has close to the ancestral chromosome number (*n* = 23), its genome size of 3.8 pg/1C differs little from some of the species with the lowest number, *n* = 15 (clade XVIII) with up to 3.8 pg/1C.

It is clear that altered repeat content is driving genome size changes in this group. However, chromosome number change is not correlated with the direction of genome size alteration in a systematic manner. Previous studies have shown that relative to parental diploids, e.g. in *N. tabacum* (a relatively recently formed allotetraploid, >100 000 years ago), there is reduced content of several repeat sequences, including tandem repeats (Lim *et al.*, 2004; Koukalova *et al.*, 2010; Renny-Byfield *et al.*, 2012), pararetroviral (Gregor *et al.*, 2004) and geminivirus-like (Skalicka *et al* 2005) sequences and various retrotransposons (Melayah *et al.*, 2004; Petit *et al.*, 2007), frequently from the paternal genome (Mhiri *et al.*, 2019). In another recently formed allotetraploid, *N. rustica*, the NPAMBO repeat was reduced by at least 10-fold compared with the maternal donor species, *N. paniculata*, which could have contributed to the observed 2–5 % reduction in DNA amount in this species (Leitch *et al.*, 2008). In the case of the species of *N.* sect. *Suaveolentes*, assessments of repeat content relative to their diploid parents is made difficult by the antiquity of the group (if they appeared 6 Mya, then there is 12 million years of divergence that separates these species from the modern relatives of their parents) and the complexity of their maternal parent, which is likely to have been a diploid hybrid between species in at least two sections of the genus, perhaps *Nicotiana* sects *Alatae* and *Noctiflorae* (Kelly *et al.*, 2013; Schiavinato *et al.*, 2019).

In *Oryza*, the genome of *O. brachyantha* (a wild rice species) is 68 % smaller than that of cultivated *O. sativa*, with the larger cultivated rice genome being associated with the amplification of long terminal repeat (LTR) retrotransposons (Chen *et al.*, 2013). Only 70 % of these two genomes were collinear, with non-homologous end-joining after double-strand breakage accounting for most movements of genes. Such rearrangements could generate reproductive barriers and perhaps lead to speciation if disruptive selection was also operating. It is likely that genome size change and chromosome rearrangements in species of *N.* sect. *Suaveolentes* could also be creating interspecific reproductive barriers.

Interspecific hybridization and allopolyploidization can trigger activation of (retro)transposons (Parisod *et al.*, 2010), as can environmental stress (Grandbastien *et al.*, 2005), both potentially triggering chromosome number and/or genome size change. However, given the lag phase between *N.* sect. *Suaveolentes* formation (6 Mya) and species radiation (2 Mya) into the arid zone, stress may have been significant to the changes observed. Overall, the ecological and evolutionary features associated with speciation in the harsh conditions of the arid zone in Australia certainly favour chromosome reduction in line with the ideas of Stebbins (1950) and earlier by Darlington (1937). These could be expected to happen in parallel both within and between clades, which we see happening independently in several clades, perhaps in as many as four (Fig. 4): *N. benthamiana/karijini* (clade VII); *N. truncata/excelsior* (clade XIV); the *N.velutina* clade (XVII); and the *N. suaveolens* clade (XVII). We hypothesize that decreases are the most likely direction of change in this group, despite the appearance of putative increases in a few cases (i.e. *N. goodspeedii*). Consequently, the optimization of chromosome number change that does not permit increases was modelled (Fig. 4C), even though we admit that it is not the simplest explanation. Further study should be able to clarify this topic, and our preference for number reduction does not influence any of our general conclusions below about chromosome number and genome size change.

### Recombination, chromosome number and genome size change

An explanation for increasing genome size observed here in the species with the lowest chromosome numbers might be that with chromosome number decreasing, the recombination rate falls. Assuming chiasma frequency equates with frequency of homologous recombination-based removal of repeats per chromosome complement, the competing rates of repeat increase/elimination reach a tipping point when the recombination rate can no longer compensate for the rate at which repeats are multiplying. This results in the overall repeat content of a genome increasing, leading to larger genome sizes overall. This hypothesis is entirely mechanistic and is the result of intrinsic repeat expansion rates vs. excision rates via recombination.

In the *n* = 15–19 species of *N.* sect. *Suaveolentes* (Fig. 4B, C), genome size increases relative to those with *n* = 20–21 (*N. heterantha*, *n* = 24 and 2.5 pg/1C, being an obvious exception to these patterns of genome size change). Except for *N. burbidgeae* (clade VIII), clades XI–XV (*n* = 20–23) have genome sizes that are 2.7–3.0 pg/1C, whereas those in clades VII, XIV, XVII and XVIII (*n* = 15–19) vary between 3.3 and 3.8 pg, an increase of 10–29 %. Potentially, when chromosome number has dropped far enough to reach an inflexion point, in this case <20 pairs of chromosomes, the number of chiasma per chromosome complement, typically 1-2 chiasma per bivalent (Goodspeed, 1954), dwindles to the point at which genome size increases due to inefficient removal via recombination. We do not expect that this specific number of chromosomes should universally cause this sort of change because it would depend on many different components that govern types and distributions of repeats, the overall genome size range (i.e. Mbp or Gbp) and other factors including population sizes and breeding systems. It also introduces a more general paradox that deserves much more attention: how have the small genomes of annual herbaceous species such as *Arabidopsis thaliana* become associated with only five pairs of chromosomes?

We hypothesize that the key to understanding this paradox might be in the phenomenon we observe here in *N. heterantha* (*n* = 24), which has the smallest genome observed in *N.* sect. *Suaveolentes*, even smaller than the South American diploids from which the section was derived. Perhaps because it has more chromosomes and is thus highly efficient in eliminating retrotransposons, it could establish a new starting small genome size that leads in its offspring to even smaller genomes during chromosome number diploidization than those observed in extant species of *Nicotiana*. Such downward genome size leaps could be important in the ancestry of species with the smallest genomes, despite their possession of only a few chromosomes, which should be associated with increasing genome size, as observed here in *Nicotiana*. A similar pattern has been observed in Brassicaceae tribes Physarieae and Anchonieae (Lysak *et al.*, 2009) and Caricaceae (Rockinger *et al.*, 2016). In the orchid species in *Erycina* (Oncidiinae; Chase *et al.*, 2005), which has the lowest number of chromosomes in Orchidaceae (*n* = 5, 7) and a smaller genome size than most orchids, genome size decreased first in the clades in which this genus is embedded, which have many chromosomes (*n* = 28, 30). Thus, genome size first decreased in species with many chromosomes and, once it was small, dramatic chromosome decreases took place, after which genome size began to increase in *Erycina* and its relative *Tolumnia* (Chase *et al.*, 2005). In *Genlisea* (Fleischmann *et al.*, 2014), it is the polyploids that exhibit the smallest genome sizes. Based on what we have observed here and these examples from the literature, the lower rates of recombination in species with fewer chromosomes should allow genome size to increase, and it is only through stochastic leaps to smaller genome sizes in species with more chromosomes that massively smaller genome sizes evolve. In this model, major reductions in genome size occur before chromosome number changes. Importantly, it is at the species and population interface that we should expect to find answers to questions regarding which factors induce genome size and chromosome number changes, and what principles govern their interactions.

### Conclusions and prospects

Based on previous literature (as reviewed in the Introduction), there is little foundation to our expectation that chromosome number changes in parallel with genome size variation, although it might be expected that as genomes are re-arranged during the formation of a descending dysploid series there would be genome size change (i.e. rates of change in the two are correlated in spite of the directions not being parallel), a situation that we do observe in *N.* sect. *Suaveolentes*. Genome size in the species of *N*. sect. *Suaveolentes* both decreases and increases as chromosome numbers decrease (Fig. 5), and in one case (*N. heterantha*, *n* = 24, 2.5 pg; Table 1) genome size has decreased drastically with no change from the ancestral chromosome number.

If chromosome rearrangements result in reduced introgression for genes carried in the re-organized chromosome arms, we expect to see greater phylogenetic concordance in loci from rearranged than from non-rearranged chromosome arms, and these should also have earlier coalescence than those in parts of the genome still experiencing gene flow. When combined with a detailed karyotypic study, this permits us to ask if different models better fit chromosome segments with varying histories, allowing us to detect distinctive evolutionary dynamics and ultimately to piece together the general history of speciation in this group. If rearrangements instigate divergence, then we expect the times at which they are established to coincide with speciation events; however, if they occur afterwards, this coincidence would not be discovered. Using coalescent models to date multiple speciation events and then mapping rearrangements on these species trees will help determine if changes in chromosome structure (number) have generally been involved in, but are not necessarily driving, speciation (Faria and Navarro, 2010). The key here will be finding systems in which chromosome change is relatively recent so that we can distinguish between the effects of disruptive selection (genic divergence), gene sweeps and changes in genomic architecture on population divergence and speciation. *Nicotiana* sect. *Suaveolentes* has many of the attributes of such a system.

## SUPPLEMENTARY DATA

Supplementary data are available online at https://academic.oup.com/aob and consist of Figure S1: Chromosome number evolution as estimated with ChromEvol with increases and decreases equally likely.

mcac006_suppl_Supplementary_Figure_S1

mcac006_suppl_Supplementary_Figure_Legend

## References

[CIT0001] Baird NA, Etter PD, Atwood TS, et al. 2008. Rapid SNP discovery and genetic mapping using sequenced RAD markers. PLoS One 3: e3376. 18852878 10.1371/journal.pone.0003376 PMC2557064

[CIT0002] Bally J, Marks C, Jung H, et al. 2021. Nicotiana paulineana, a new Australian species in Nicotiana section Suaveolentes. Australian Systematic Botany 34: 477–484.

[CIT0003] Bennetzen JL, Kellogg EA. 1997. Do plants have a one-way ticket to genomic obesity. The Plant Cell 9: 1509–1514. 12237393 10.1105/tpc.9.9.1509 PMC157029

[CIT0004] Blomberg SP, Lefevre JG, Wells JA, Waterhouse M. 2012. Independent contrasts and PGLS regression estimators are equivalent. Systematic Biology 61: 382–391. 22215720 10.1093/sysbio/syr118

[CIT0005] Bombarely A, Rosli HG, Vrebalov J, Moffett P, Mueller LA, Martin GB. 2012. A draft genome sequence of *Nicotiana benthamiana* to enhance molecular plant–microbe biology research. Molecular Plant-Microbe Interactions 25: 1523–1530. 22876960 10.1094/MPMI-06-12-0148-TA

[CIT0006] Brandrud MK, Paun O, Lorenz R, Baar J, Hedrén M. 2019. Restriction-site associated DNA sequencing supports a sister group relationship of *Nigritella* and *Gymnadenia* (Orchidaceae). Molecular Phylogenetics and Evolution 136: 21–28. 30914398 10.1016/j.ympev.2019.03.018 PMC7613184

[CIT0007] Brandrud MK, Baar J, Lorenzo MT, et al. 2020. Phylogenomic relationships of diploids and the origins of allotetraploids in *Dactylorhiza* (Orchidaceae). Systematic Biology 69: 91–109. 31127939 10.1093/sysbio/syz035 PMC6902629

[CIT0008] Burbidge N . 1960. The Australian species of *Nicotiana* L. (Solanaceae). Australian Journal of Botany 8: 342–378.

[CIT0009] Byrne M, Yeates DK, Joseph L, et al. 2008. Birth of a biome: insights into the assembly and maintenance of the Australian arid zone biota. Molecular Ecology 17: 4398–4417. 18761619 10.1111/j.1365-294X.2008.03899.x

[CIT0010] Carlquist S . 2009. Xylem heterochrony: an unappreciated key to angiosperm origin and diversification. Botanical Journal of the Linnean Society 161: 26–65.

[CIT0011] Carta A, Bedini G, Peruzzi L. 2018. Unscrambling phylogenetic effects and ecological determinants of chromosome number in major angiosperm clades. Scientific Reports 8: 1–14. 30250220 10.1038/s41598-018-32515-x PMC6155329

[CIT0012] Carta A, Bedini G, Peruzzi L. 2020. A deep dive into the ancestral chromosome number and genome size of flowering plants. New Phytologist 228: 1097–1106. 32421860 10.1111/nph.16668

[CIT0013] Cauz-Santos LA, Hohenlohe PA, Bassham S, Amores A, Cresko WA. 2013. Stacks: an analysis tool set for population genetics. Molecular Ecology 22: 3124–3140. 23701397 10.1111/mec.12354 PMC3936987

[CIT0013a] Cauz-Santos LA, Dodsworth S, Samuel R, Christenhusz MJM, Patel D, Shittu T, Jakob A, Paun O, Chase MW. 2022. Variation of the Rdr1 gene insertion in wild populations of Nicotiana benthamiana (Solanaceae) and insights into recent species divergence. BioRxiv doi: 10.1101/2022.02.01.478068 PMC9543217 35535507

[CIT0014] Chase MW, Christenhusz MJM. 2018 *a*. Nicotiana stenocarpa. Curtis’s Botanical Magazine 35: 318–327.

[CIT0015] Chase MW, Christenhusz MJM. 2018 *b*. Nicotiana karijini. Curtis’s Botanical Magazine 35: 228–236.

[CIT0016a] Chase MW, Christenhusz MJM. 2018 *c*. Nicotiana gascoynica. Curtis’s Botanical Magazine 35: 245–252.

[CIT0017] Chase MW, Christenhusz MJM. 2021 *a*. Nicotiana insecticida. Curtis’s Botanical Magazine 38: 350–364.

[CIT0018] Chase MW, Christenhusz MJM. 2021 *b*. Nicotiana pila. Curtis’s Botanical Magazine 38: 394–404.

[CIT0020] Chase MW, Knapp S, Cox AV, et al. 2003. Molecular systematics, GISH and the origin of hybrid taxa in *Nicotiana* (Solanaceae). Annals of Botany 92: 107–127. 12824072 10.1093/aob/mcg087 PMC4243627

[CIT0021] Chase MW, Hanson L, Albert VA, Whitten MW, Williams NH. 2005. Life history evolution and genome size in subtribe Oncidiinae (Orchidaceae). Annals of Botany 95: 191–199. 15596466 10.1093/aob/mci012 PMC4246717

[CIT0022] Chase MW, Christenhusz MJM, Conran JG, et al. 2018 *a*. Unexpected diversity of Australian tobacco species (*Nicotiana* section *Suaveolentes*, Solanaceae). Curtis’s Botanical Magazine 35: 212–227.

[CIT0023] Chase MW, Conran JG, Christenhusz MJM. 2018 *b*. Nicotiana yandinga. Curtis’s Botanical Magazine 35: 237–244.

[CIT0024] Chase MW, Conran JG, Christenhusz MJM. 2018 *c*. Nicotiana faucicola. Curtis’s Botanical Magazine 35: 253–260.

[CIT0025] Chase MW, Christenhusz MJM, Palsson RL, et al. 2021 *a*. Species delimitation in *Nicotiana* sect. *Suaveolentes* (Solanaceae): reciprocal illumination leads to recognition of many new species. Curtis’s Botanical Magazine 38: 266–286.

[CIT0026] Chase MW, Dodsworth S, Christenhusz MJM. 2021 *b*. Nicotiana walpa. Curtis’s Botanical Magazine 38: 298–308.

[CIT0027a] Chase MW, Fay MF, Christenhusz MJM. 2021 *c*. Nicotiana salina. Curtis’s Botanical Magazine 38: 416–424.

[CIT0028] Chase MW, Fay MF, Nollet F, Christenhusz MJM. 2021 *d*. Nicotiana notha. Curtis’s Botanical Magazine 38: 340–349.

[CIT0029] Chen J, Huang Q, Gao D, et al. 2013. Whole-genome sequencing of *Oryza brachyantha* reveals mechanisms underlying *Oryza* genome evolution. Nature Communications 4: 1595. 10.1038/ncomms2596 PMC3615480 23481403

[CIT0030] Clarkson JJ, Knapp S, Garcia V, Olmstead RG, Chase MW. 2004. Phylogenetic relationships in *Nicotiana* (Solanaceae) inferred from multiple plastid DNA regions. Molecular Phylogenetics and Evolution 33: 75–90. 15324840 10.1016/j.ympev.2004.05.002

[CIT0031] Clarkson JJ, Kelly LJ, Leitch AR, Knapp S, Chase MW. 2010. Nuclear glutamine synthetase evolution in *Nicotiana*: phylogenetics and the origins of allotetraploid and homoploid (diploid) hybrids. Molecular Phylogenetics and Evolution 55: 95–112. 10.1016/j.ympev.2009.10.003 19818862

[CIT0032] Clarkson JJ, Dodsworth S, Chase MW. 2017. Time-calibrated phylogenetic trees establish a lag phase between polyploidisation and diversification in *Nicotiana* (Solanaceae). Plant Systematics and Evolution 303: 1001–1021. 10.1007/s00606-016-1356-9 PMC6961477 32009724

[CIT0033] Cruaud A, Gautier M, Galan M, et al. 2014. Empirical assessment of RAD sequencing for interspecific phylogeny. Molecular Biology and Evolution 31: 1272–1274. 24497030 10.1093/molbev/msu063

[CIT0034] Danecek P, Auton A, Abecasis G, et al. 2011. The variant call format and VCFtools. Bioinformatics 27: 2156–2158. 21653522 10.1093/bioinformatics/btr330 PMC3137218

[CIT0035] Darlington CD . 1937. Recent advances in cytology, 2nd edn. London: Churchill.

[CIT0036] Dodsworth S, Chase MW, Leitch AR. 2015. Is post-polyploidization diploidization the key to the evolutionary success of angiosperms? Botanical Journal of the Linnean Society 180: 1–5.

[CIT0037] Dodsworth S, Guignard MS, Pérez-Escobar OA, Struebig M, Chase MW, Leitch AR. 2020 *a*. Repetitive DNA restructuring across multiple *Nicotiana* allopolyploidisation events shows a lack of strong cytoplasmic bias in influencing repeat turnover. Genes 11: 216. 32092894 10.3390/genes11020216 PMC7074350

[CIT0038] Dodsworth S, Christenhusz MJM, Conran JG, et al. 2020 *b*. Extensive plastid–nuclear discordance in a recent radiation of *Nicotiana* section *Suaveolentes* (Solanaceae). Botanical Journal of the Linnean Society 193: 546–559.

[CIT0039] Doyle JJ . 1990. Isolation of plant DNA from fresh tissue. Focus 12: 13–15.

[CIT0040] Escudero M, Wendel JF. 2020. The grand sweep of chromosomal evolution in angiosperms. New Phytologist 228: 805–808. 32772369 10.1111/nph.16802

[CIT0041] Faria R, Navarro A. 2010. Chromosomal speciation revisited: rearranging theory with pieces of evidence. Trends in Ecology and Evolution 25: 660–669. 20817305 10.1016/j.tree.2010.07.008

[CIT0042] Fedoroff NV . 2012. Transposable elements, epigenetics, and genome evolution. Science 338: 758–767. 23145453 10.1126/science.338.6108.758

[CIT0043] Fleischmann A, Michael TP, Rivadavia F, et al. 2014. Evolution of genome size and chromosome number in the carnivorous plant genus *Genlisea* (Lentibulariaceae), with a new estimate of the minimum genome size in angiosperms. Annals of Botany 114: 1651–1663. 25274549 10.1093/aob/mcu189 PMC4649684

[CIT0044] Garland T, Harvey PH, Ives AR. 1992. Procedures for the analysis of comparative data using phylogenetically independent contrasts. Systematic Biology 41: 18–32.

[CIT0045] Glick L, Mayrose I. 2014. ChromEvol: assessing the pattern of chromosome number evolution and the inference of polyploidy along a phylogeny. Molecular Biology and Evolution 31: 1914–1922. 24710517 10.1093/molbev/msu122

[CIT0046] Goldblatt P, Johnson DE. 1979. *Index to plant chromosome numbers (IPCN).* http://legacy.tropicos.org/Project/IPCN.

[CIT0047] Goodspeed TH . 1954. The genus Nicotiana: origins, relationships and evolution of its species in the light of their distributions, morphology and cytogenetics. Waltham, MA: Chronica Botanica.

[CIT0048] Grandbastien M, Audeon C, Bonnivard E, et al. 2005. Stress activation and genomic impact of *Tnt1* retrotransposons in Solanaceae. Cytogenetic and Genome Research 110: 229–241. 16093677 10.1159/000084957

[CIT0049] Gregor W, Mette MF, Staginnus C, Matzke MA, Matzke AJM. 2004. A distinct endogenous pararetrovirus family in *Nicotiana tomentosiformis*, a diploid progenitor of polyploid tobacco. Plant Physiology 134: 1191–1199. 14988473 10.1104/pp.103.031112 PMC389943

[CIT0050] Grover CE, Wendel JF. 2010. Recent insights into mechanisms of genome size change in plants. Journal of Botany 2010: 382732.

[CIT0051] Hawkins JS, Proulx SR, Rapp RA, Wendel JF. 2009. Rapid DNA loss as a counterbalance to genome expansion through retrotransposon proliferation in plants. Proceedings of the National Academy of Sciences, USA 106: 17811–17816. 10.1073/pnas.0904339106 PMC2764891 19815511

[CIT0052] Heckenhauer J, Samuel R, Aston PS, Salim KA, Paun O. 2018. Phylogenomics resolves evolutionary relationships and provides insights into floral evolution in the tribe Shoreeae. Molecular Phylogenetics and Evolution 127: 1–13. 29778722 10.1016/j.ympev.2018.05.010

[CIT0053] Horton P . 1981. A taxonomic revision of *Nicotiana* (Solanaceae) in Australia. Journal of the Adelaide Botanical Garden 3: 1–56.

[CIT0054] Hufton AL, Panopoulou G. 2009. Polyploidy and genome restructuring: a variety of outcomes. Current Opinion in Genetics & Development 19: 600–606. 19900800 10.1016/j.gde.2009.10.005

[CIT0055] Kelly LJ, Leitch AR, Clarkson JJ, Knapp S, Chase MW. 2013. Reconstructing the complex evolutionary origin of wild allopolyploid tobaccos (*Nicotiana* section *Suaveolentes*). Evolution 76: 80–94. 10.1111/j.1558-5646.2012.01748.x 23289563

[CIT0057] Kitamura S, Tanaka A, Inoue M. 2005. Genomic relationships among *Nicotiana* species with different ploidy levels revealed by 5S rDNA spacer sequences and FISH/GISH. Genes and Genetic Systems 80: 251–260. 16284418 10.1266/ggs.80.251

[CIT0058] Korneliussen TS, Albrechtsen A, Nielsen R. 2014. ANGSD: analysis of next generation sequencing data. BMC Bioinformatics 15: 356. 25420514 10.1186/s12859-014-0356-4 PMC4248462

[CIT0059] Koukalova B, Moraes AP, Renny-Byfield S, Matyasek R, Leitch AR, Kovarik A. 2010. Fall and rise of satellite repeats in allopolyploids of *Nicotiana* over c. 5 million years. New Phytologist 186: 148–160. 19968801 10.1111/j.1469-8137.2009.03101.x

[CIT0060] Leitch AR, Leitch IJ. 2008. Genomic plasticity and the diversity of polyploid plants. Science 320: 481–483. 18436776 10.1126/science.1153585

[CIT0061] Leitch IJ, Bennett MD. 2004. Genome down-sizing in polyploid plants. Botanical Journal of the Linnean Society 82: 651–663.

[CIT0062] Leitch IJ, Hanson L, Lim KY, et al. 2008. The ups and downs of genome size evolution in polyploid species of *Nicotiana* (Solanaceae). Annals of Botany 101: 805–814. 18222910 10.1093/aob/mcm326 PMC2710205

[CIT0063] Lewis PO . 2001. A likelihood approach to estimating phylogeny from discrete morphological character data. Systematic Biology 50: 913–925. 12116640 10.1080/106351501753462876

[CIT0064] Li H, Durbin R. 2009. Fast and accurate short read alignment with Burrows–Wheeler transform. Bioinformatics 25: 1754–1760. 19451168 10.1093/bioinformatics/btp324 PMC2705234

[CIT0065] Lim KY, Matyasek R, Kovarik A, Leitch AR. 2004. Genome evolution in allotetraploid Nicotiana. Biological Journal of the Linnean Society 82: 599–606.

[CIT0066] Lischer HE, Excoffier L. 2012. PGDSpider: an automated data conversion tool for connecting population genetics and genomics programs. Bioinformatics 28: 298–299. 22110245 10.1093/bioinformatics/btr642

[CIT0067] Loureiro J, Rodriguez E, Doležel J, Santos C. 2007. Two new nuclear isolation buffers for plant DANN flow cytometry: a test with 37 species. AoB Plants 100: 875–888. 10.1093/aob/mcm152 PMC2749623 17684025

[CIT0068] Lysak MA, Koch MA, Beaulieu JM, Meister A, Leitch IJ. 2009. The dynamic ups and downs of genome size evolution in Brassicaceae. Molecular Biology and Evolution 26: 85–98. 18842687 10.1093/molbev/msn223

[CIT0069] Maas DL, Prost S, Bi K, et al. 2018. Rapid divergence of mussel populations despite incomplete barriers to dispersal. Molecular Ecology 27: 1556–1571. 29575349 10.1111/mec.14556

[CIT0070] Mandáková T, Lysak MA. 2008. Chromosomal phylogeny and karyotype evolution in *x* = 7 crucifer species (Brassicaceae). The Plant Cell 20: 2559–2570. 18836039 10.1105/tpc.108.062166 PMC2590746

[CIT0071] Marks CE, Newbigin E, Ladiges PY. 2011. Comparative morphology and phylogeny of *Nicotiana* section *Suaveolentes* (Solanaceae) in Australia and the South Pacific. Australian Systematic Botany 24: 61–86.

[CIT0072] McKenna A, Hanna M, Banks E, et al. 2010. The Genome Analysis Toolkit: a MapReduce framework for analyzing next-generation DNA sequencing data. Genome Resources 9: 1297–1303. 10.1101/gr.107524.110 PMC2928508 20644199

[CIT0073] Meisner J, Albrechtsen A. 2018. Inferring population structure and admixture proportions in low-depth NGS data. Genetics 210: 719–731. 30131346 10.1534/genetics.118.301336 PMC6216594

[CIT0074] Melayah D, Lim KY, Bonnivard E, et al. 2004. Distribution of the *Tnt1* retrotransposon family in the amphidiploid tobacco (*Nicotiana tabacum*) and its wild *Nicotiana* relatives. Biological Journal of the Linnean Society 82: 639–649.

[CIT0075] Merot C, Oomen RA, Tigano A, Wellenreuther M. 2020. A roadmap for understanding the evolutionary significance of structural genomic variation. Trends in Ecology & Evolution 35: 561–572. 32521241 10.1016/j.tree.2020.03.002

[CIT0076] Mhiri C, Parisod C, Daniel J, et al. 2019. Parental transposable element loads influence their dynamics in young *Nicotiana* hybrids and allotetraploids. New Phytologist 221: 1619–1633. 30220091 10.1111/nph.15484

[CIT0077] Michael TP . 2014. Plant genome size variation: bloating and purging DNA. Briefings in Functional Genomics 13: 308–317. 24651721 10.1093/bfgp/elu005

[CIT0078] Morjan CL, Rieseberg LH. 2004. How species evolve collectively: implications of gene flow and selection for the spread of advantageous alleles. Molecular Ecology 13: 1341–1356. 15140081 10.1111/j.1365-294X.2004.02164.x PMC2600545

[CIT0079] Narayan RKJ . 1987. Nuclear DNA changes, genome differentiation and evolution in *Nicotiana* (Solanaceae). Plant Systematics and Evolution 157: 161–180.

[CIT0080] Oliver KR, Greene WK. 2009. Transposable elements: powerful facilitators of evolution. BioEssays 31: 703–714. 19415638 10.1002/bies.200800219

[CIT0081] Oliver KR, McCom JA, Greene WK. 2009. Transposable elements: powerful contributors to angiosperms evolution and diversity. Genome Biology and Evolution 5: 1886–1901. 10.1093/gbe/evt141 PMC3814199 24065734

[CIT0082] Ortiz-Barrientos D, Engelstädter J, Rieseberg LH. 2016. Recombination rate evolution and the origin of species. Trends in Ecology and Evolution 31: 226–236. 26831635 10.1016/j.tree.2015.12.016

[CIT0083] Pagel M, Meade A, Barker D. 2004. Bayesian estimation of ancestral character states on phylogenies. Systematic Biology 53: 673–684. 15545248 10.1080/10635150490522232

[CIT0084] Pagès H, Aboyoun P, Gentleman R, DebRoy S. 2020. Biostrings: efficient manipulation of biological strings. R package version 2.56.0. https://bioconductor.org/packages/Biostrings

[CIT0085] Paradis E, Schliep K. 2018. ape 5.0: an environment for modern phylogenetics and evolutionary analyses in R. Bioinformatics 35: 526–528. 10.1093/bioinformatics/bty633 30016406

[CIT0086] Parisod C, Alix K, Jusst J, et al. 2010. Impact of transposable elements on the organization and function of allopolyploid genomes. New Phytologist 186: 37–45. 20002321 10.1111/j.1469-8137.2009.03096.x

[CIT0087] Paun O, Turner B, Trucchi E, Munzinger J, Chase MW, Samuel R. 2016. Processes driving the adaptive radiation of a tropical tree (*Diospyros*, Ebenaceae) in New Caledonia, a biodiversity hotspot. Systematic Biology 65: 212–227. 26430059 10.1093/sysbio/syv076 PMC4748748

[CIT0088] Pedersen TL . 2020. *patchwork: the composer of plots.* https://CRAN.Rproject.org/package=patchwork.

[CIT0089] Pellicer J, Leitch IJ. 2014. The application of flow cytometry for estimating genome size and ploidy level in plants. Methods in Molecular Biology 1115: 279–307. 24415480 10.1007/978-1-62703-767-9_14

[CIT0090] Petit M, Lim K, Julio E, et al. 2007. Differential impact of retrotransposon populations on the genome of allotetraploid tobacco (*Nicotiana tabacum*). Molecular Genetics and Genomics 278: 1–15. 17375323 10.1007/s00438-007-0226-0

[CIT0091] Potter S, Bragg JG, Blom MP, et al. 2017. Chromosomal speciation in the genomics era: disentangling phylogenetic evolution of rock-wallabies. Frontiers in Genetics 10:10. 10.3389/fgene.2017.00010 PMC5301020 28265284

[CIT0092] Purdie RW, Symon DE, Haegi L. 1982. Nicotiana. In: George AS, ed. Flora of Australia, 29. Canberra: Australian Government Publishing Service.

[CIT0093] Puttick MN, Clark J, Donoghue PCJ. 2015. Size is not everything: rates of genome size evolution, not C-value, correlate with speciation in angiosperms. Proceedings of the Royal Society B: Biological Sciences 282: 20152289. 10.1098/rspb.2015.2289 PMC4685785 26631568

[CIT0094] Renny-Byfield S, Kovařík A, Chester M, et al. 2012. Independent, rapid and targeted loss of highly repetitive DNA in natural and synthetic allopolyploids of Nicotiana tabacum. PLoS One 7: e3696. 10.1371/journal.pone.0036963 PMC3351487 22606317

[CIT0095] Rieseberg LH . 2001. Chromosomal rearrangements and speciation. Trends in Ecology and Evolution 16: 351–358. 11403867 10.1016/s0169-5347(01)02187-5

[CIT0096] Robertson FM, Gundappa MK, Grammes F, et al. 2017. Lineage-specific rediploidization is a mechanism to explain time-lags between genome duplication and evolutionary diversification. Genome Biology 18: 1–4. 28615063 10.1186/s13059-017-1241-z PMC5470254

[CIT0097] Rockinger A, Sousa A, Carvalho FA, Renner SS. 2016. Chromosome number reduction in the sister clade of *Carica* papaya with concomitant genome size doubling. American Journal of Botany 103: 1082–1088. 27234227 10.3732/ajb.1600134

[CIT0098] Rupp BR, Samuel R, Russell A, Temsch E, Chase MW, Leitch I. 2010. Genome size in *Polystachya* (Orchidaceae) and its relationship to epidermal characters. Botanical Journal of the Linnean Society 163: 223–233.

[CIT0099] Schiavinato M, Marcet-Houben M, Dohm JC, Gabaldon T, Himmelbauer H. 2019. Parental origin of the allotetraploid tobacco Nicotiana benthamiana. The Plant Journal 102: 541–554. 10.1111/tpj.14648 PMC7317763 31833111

[CIT0100] Schubert I, Vu GTH. 2016. Genome stability and evolution: attempting a holistic view. Trends in Plant Science 21: 749–757. 27427334 10.1016/j.tplants.2016.06.003

[CIT0101] Schweizer D . 1976. Reverse fluorescent chromosome banding with chromomycin and DAPI. Chromosoma 58: 307–324. 137107 10.1007/BF00292840

[CIT0102] Skalicka K, Lim KY, Matyasek R, Matzke M, Leitch AR, Kovarik A. 2005. Preferential elimination of repeated DNA sequences from the paternal, *Nicotiana tomentosiformis* genome donor of a synthetic, allotetraploid tobacco. New Phytologist 166: 291–303. 15760371 10.1111/j.1469-8137.2004.01297.x

[CIT0103] Soltis DE, Visger CJ, Marchant DB, Soltis PS. 2016. Polyploidy: pitfalls and paths to a paradigm. American Journal of Botany 103: 1146–1166. 27234228 10.3732/ajb.1500501

[CIT0104] Stamatakis A . 2014. RAxML version 8: a tool for phylogenetic analysis and post-analysis of large phylogenies. Bioinformatics 30: 1312–1313. 24451623 10.1093/bioinformatics/btu033 PMC3998144

[CIT0105] Stebbins GL . 1950. Variation and evolution in plants. New York: Columbia University Press.

[CIT0106] Symon DE . 1984. A new species of *Nicotiana* (Solanaceae) from Dalhousie Springs, South Australia. Journal of the Adelaide Botanical Garden 7: 117–121.

[CIT0107] Symon DE . 1998. A new *Nicotiana* (Solanaceae) from near Coober Pedy, South Australia. Journal of the Adelaide Botanical Garden 18: 1–4.

[CIT0108] Tatemichi Y . 1990. Illustrated book of the genus Nicotiana. Toyoda: Japan Tobacco Company.

[CIT0109] Tregoning JS, Brown ES, Cheeseman HM, et al. 2020. Vaccines for COVID‐19. Clinical and Experimental Immunology 202: 162–192. 32935331 10.1111/cei.13517 PMC7597597

[CIT0110] Van der Knaap E, Sanyal A, Jackson SA, Tanksley SD. 2004. High-resolution fine mapping and fluorescence *in situ* hybridization analysis of *sun*, a locus controlling tomato fruit shape, reveals a region of the tomato genome prone to DNA rearrangements. Genetics 168: 2127–2140. 15611181 10.1534/genetics.104.031013 PMC1448703

[CIT0111] Wagner ND, He L, Hörandl E. 2020. Phylogenomic relationships and evolution of polyploid *Salix* species revealed by RAD sequencing data. Frontiers in Plant Sciences 11: 1077. 10.3389/fpls.2020.01077 PMC7379873 32765560

[CIT0112] Wang L, Lam TT, Xu S, et al. 2020. treeio: an R package for phylogenetic tree input and output with richly annotated and associated data. Molecular Biology and Evolution 37: 599–603. 31633786 10.1093/molbev/msz240 PMC6993851

[CIT0113] Wang X, Morton J, Pellicer J, Leitch IJ, Leitch AR. 2021. Genome downsizing after polyploidy: mechanisms, rates and selection pressures. The Plant Journal 107: 1003–1015. 34077584 10.1111/tpj.15363

[CIT0114] Warmuth VM, Ellegren H. 2019. Genotype-free estimation of allele frequencies reduces bias and improves demographic inference from RADseq data. Molecular Ecology Resources 19: 586–596. 30633448 10.1111/1755-0998.12990

[CIT0115] Warnes GR, Bolker B, Bonebakker L, et al. 2020. gplots: various R programming tools for plotting data. R package version 3. https://github.com/talgalili/gplots

[CIT0116] Wendel JF . 2015. The wondrous cycles of polyploidy in plants. American Journal of Botany 102: 1753–1756. 26451037 10.3732/ajb.1500320

[CIT0117] Wickham H . 2016. ggplot2: *elegant graphics for data analysis*. New York: Springer.

[CIT0118] Williams E . 1975. A new chromosome number in the Australian species *Nicotiana cavicola* L. (Burbidge). New Zealand Journal of Botany 13: 11–12.

[CIT0119] Yu G . 2020. Using ggtree to visualize data on tree-like structures. Current Protocols in Bioinformatics 69: e96. 32162851 10.1002/cpbi.96

[CIT0120] Yu G, Smith DK, Zhu H, Guan Y, Lam TTY. 2017. ggtree: an R package for visualization and annotation of phylogenetic trees with their covariates and other associated data. Methods in Ecology and Evolution 8: 28–36.

