# Supplementary material for: Down, then up: non-parallel genome size changes and a descending chromosome series in a recent radiation of the Australian allotetraploid plant species, Nicotiana section Suaveolentes (Solanaceae)
Source: Ann Bot. 2022 Jan 13;131(1):123–42. doi: 10.1093/aob/mcac006 (PMC9904355; doi:10.1093/aob/mcac006)
Supplement: mcac006_suppl_Supplementary_Figure_S1 [file mcac006_suppl_supplementary_figure_s1.pdf]

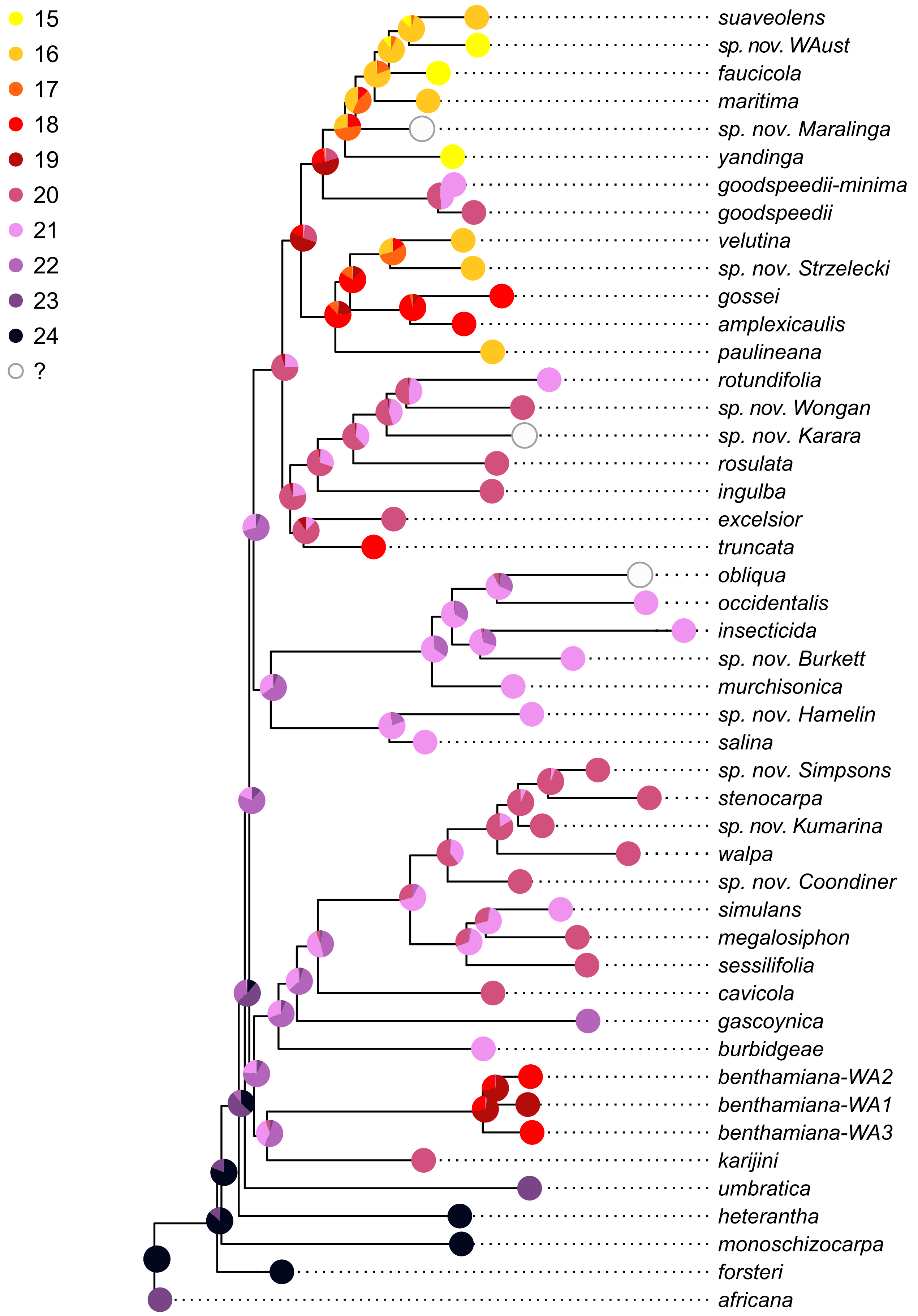

Supplementary Figure 1. Present state and ancestral reconstruction of chromosome number. The summary tree of species relationships (with locality names for undescribed new species, as in Fig. 3), as estimated with ChromEvol with chromosome number increases and decreases given equal probabilities.
