# Supplementary material for: Down, then up: non-parallel genome size changes and a descending chromosome series in a recent radiation of the Australian allotetraploid plant species, Nicotiana section Suaveolentes (Solanaceae)
Source: Ann Bot. 2022 Jan 13;131(1):123–42. doi: 10.1093/aob/mcac006 (PMC9904355; doi:10.1093/aob/mcac006)
Supplement: mcac006_suppl_Supplementary_Figure_Legend [file mcac006_suppl_supplementary_figure_legend.docx]

Supplementary data Figure caption

Figure S1. The summary tree of species relationships (with locality names for undescribed new species, as in Fig. 3), as estimated with ChromEvol in which chromosome number increases and decreases are equally likely.
